# Ligand Drift Rate Descriptor in Receptor Pocket: Molecular Docking Sampling for Differentiating Partial Agonists in Nuclear Receptors

**DOI:** 10.1021/acs.jcim.6c00669

**Published:** 2026-07-23

**Authors:** Ying-Ting Lin, Yung-Ting Shih

**Affiliations:** † Department of Biotechnology, College of Life Science, 38023 Kaohsiung Medical University, Kaohsiung 807, Taiwan; ‡ Drug Development & Value Creation Research Center, Kaohsiung Medical University, Kaohsiung 807, Taiwan

## Abstract

The recent discovery that inverse agonists bind to a secondary orthosteric site in PPARγ reveals unexpected structural complexity in this nuclear receptor. To differentiate between full and partial agonists, we hypothesize that partial agonists exhibit dynamic positional behavior, moving among alternative binding sites at equilibrium when saturated, while full agonists bind directly and consistently to the primary orthosteric site. Using a two-state model derived from the Boltzmann distribution and molecular docking (MODO) sampling, we developed a computational metric called the Ligand Drift Rate (LDR) Descriptor to differentiate full agonists (Emax ≥ 90%) from partial agonists (Emax ≤ 45%) of PPARγ by simulating ligand positional instability within the primary orthosteric binding pocket. We combined molecular docking and hydrogen bond fluctuation analysis across 28 PPARγ-ligand complexes (18 full agonists and 10 partial agonists). MODO sampling was performed with several algorithms, each tested on five carefully selected receptor-binding pockets from 356 crystal complexes. Hydrogen bonds between ligands and key residues (His323, His449, Tyr473) were analyzed at various hydrogen-bond displacement (HBD) thresholds (6–12 Å). The LDR was calculated as the percentage of the top 20-scoring poses that lacked hydrogen bonds. We found that adding constraints on pose diversity and allowing receptor flexibility can improve LDR’s ability to distinguish full from partial agonists. Finally, MODO-sampling-based LDR calculations were conducted on 1079 ChEMBL ligands (393 full agonists and 686 partial agonists). In this validation set, LDR successfully differentiated partial from full agonists (*t*-test, p = 0.00043) using the genetic algorithm with the 3B1M-KRC binding pocket and a 10 Å hydrogen-bond threshold. In QSAR applications for drug discovery, we have developed a dynamics-aware molecular descriptor, LDR, that can be a key factor in quantifying partial agonism arising from small-molecule ligand drift away from the primary orthosteric site.

## Graphical abstract

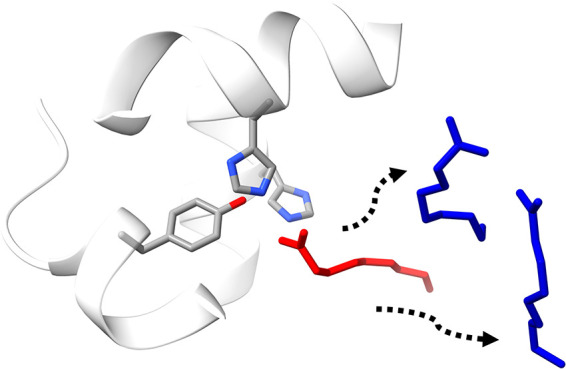

## 1 Introduction

The nuclear receptor (NR) superfamily of ligand-activated transcription factors regulates metabolism, development, and homeostasis.^1–3^ Nuclear receptors function as ligand-driven transcription factors, where small-molecule ligands act as molecular switches to regulate receptor activity and gene expression.^4^ These receptors have a modular structure consisting of an N-terminal activation function-1 (AF-1) domain, a central DNA-binding domain (DBD), and a C-terminal ligand-binding domain (LBD). The LBD features a hydrophobic ligand-binding pocket and includes the activation function-2 (AF-2) surface.^4,5^ Research indicates that numerous agonists activate NR transcription through a consistent mechanism.^4^ As a member of the NR family, PPARγ functions as a critical therapeutic target for metabolic disorders, particularly type 2 diabetes.^6–9^ Structurally diverse ligands now bind the receptor with notable flexibility, leading to diverse pharmacological effects. Full agonists, such as thiazolidinediones and rosiglitazone, bind to this primary orthosteric pocket and achieve maximum activation by stabilizing helix 12 (H12) through hydrogen bonds with residues Tyr473 (H12), His449 (H10), and His323 (H5). This stabilization promotes optimal coactivator recruitment. Antagonists may block receptor activation without inducing the conformational changes necessary for transcriptional activity.^10–12^

The recent study of the inverse agonist mechanism for peroxisome proliferator-activated receptor gamma (PPARγ) has uncovered a secondary orthosteric ligand-binding pocket within the receptor’s ligand-binding domain (LBD), fundamentally challenging the traditional view of a single orthosteric binding site.^13–17^ These inverse agonists bind to this secondary orthosteric site and actively reduce baseline receptor activity by stabilizing conformations that favor corepressor recruitment over coactivator binding. These findings broaden our understanding of nuclear receptor structural complexity, showing that different ligand types can target multiple binding regions on a conformational ensemble to produce diverse pharmacological effects. The secondary orthosteric site indicates that ligand positioning, site-specific binding, and subsequent protein recruitment patterns are key determinants of ligand activation profiles.

Given this structural complexity, distinguishing partial agonists, which produce submaximal activation, from full agonists is crucial for optimizing therapeutic efficacy while minimizing side effects. X-ray crystallography snapshots show that partial agonists can bind either to the usual orthosteric site (e.g., PDB: 2VV1, ∼2VV4) or to other binding regions (e.g., PDB: 2YFE, 2ZK2) that are inactive in the fully activated state.^18–20^ More interestingly, several PPARγ crystal snapshots (e.g., PDB: 2VSR, 4EM9) captured two or three identical ligand molecules binding simultaneously at different regions within the ligand-binding domain, with one occupying the primary orthosteric site and others at alternate sites.^18,21^ We also note that the partial agonist-bound PPARγ occupies a broad conformational energy well with multiple local minima of similar potential energy. This is supported by broad peaks in ^19^F NMR spectra, slow chemical exchange between conformational states as confirmed by CEST NMR experiments, and the sampling of multiple distinct helix 12 conformations in long-time scale molecular dynamics simulations.^17^ These empirical structural evidence suggest that partial agonists exhibit dynamic binding behavior, shifting between distinct binding sites during receptor activation. Unlike full agonists, which exclusively occupy the orthosteric site due to strong thermodynamic favorability, partial agonists distribute between the orthosteric site and other neutral sites at equilibrium, thereby limiting their maximum biological response to a fraction of a full agonist’s efficacy. In other words, a partial agonist can move away from the primary orthosteric binding site during transcription activation at a saturating concentration. Therefore, we hypothesize a two-state model that directly comes from the Boltzmann distribution. We also designed a molecular docking sampling strategy to systematically characterize agonist binding poses across multiple potential binding sites, thereby effectively mapping the energetic landscape of ligand–receptor interactions throughout the binding pocket. This approach uses explicit molecular docking simulations to thoroughly sample hydrogen-bond displacements across docking poses involving three key residues in the primary orthosteric binding site, enabling quantitative analysis of how specific small-molecule ligands exhibit dynamic binding behavior and shifting tendencies. By setting boundaries for hydrogen-bond displacements in docking poses within the primary orthosteric binding domain, we constructed a computational metric—the Ligand Drift Rate (LDR) Descriptor—to differentiate the binding-pose distribution, thus describing and predicting ligand partial agonism at the computational level.

## 2 Methods

### 2.1 Binding Pocket Selection

The molecular docking binding pockets were derived from crystallographically determined structures of PPARγ-ligand complexes in the Protein Data Bank (PDB, RCSB.org), selected based on the criteria below to ensure computational reliability.^16^ Structures were filtered from an initial collection of 356 PPARγ-ligand complexes. Complexes with the largest binding pockets (in the top 10% by volume) were prioritized to accommodate diverse ligand poses during molecular docking. We chose full agonist-bound pockets from the initial set of complexes, where the receptor–ligand conformations follow the lock-and-key binding hypothesis, providing a snapshot of the key pharmacophore that activates agonism. The receptor structures with the main ligand-occupied volume were identified as 6ENQ-BJB and were initially used to analyze the number of retained docking poses and select docking algorithms. The binding site boundaries for molecular docking were manually defined by extending 2.0 Å beyond all atoms (including hydrogens) of the cocrystallized ligand after adding hydrogens. At the same time, the primary orthosteric interaction patterns also display the AF-2 activation conformation with the proper spatial arrangement of key residues, His323, His449, and Tyr473, as observed in the crystallographic structures. After selecting GOLD’s genetic algorithm for its ability to differentiate, we systematically evaluated five PPARγ binding pockets (3B1M-KRC, 6ENQ-BJB, 6ICJ-A0L, 6ILQ-AE0, and 6T6B-MLQ) (Table 1) to identify the best receptor-binding pocket for distinguishing between full and partial agonists. These pockets were previously selected from 356 PPARγ-ligand complexes in the Protein Data Bank based on the largest binding pocket volumes (top 5 complexes). Most selected structures preserved the orthosteric AF-2 activation conformation with proper spatial arrangements of key residues His323, His449, and Tyr473.

**Table 1:** Information for the Selected Binding Pockets Is Provided, Named after the PDB Code Hyphenated with the Ligand Code^a^

Binding pocket	Chain	Emax	Resolution	Volume
6ILQ-AE0	A	114%	2.41 Å	589.5
6ENQ-BJB	A	97%	2.20 Å	358.5
6T6B-MLQ	A	96%	2.80 Å	450.125
6ICJ-A0L	A	93%	2.48 Å	521
3B1M-KRC	A	37%	1.60 Å	525.875

a Their chain ID (chain), maximum efficacy (Emax), X-ray experimental resolution (resolution), and binding pocket volume (volume) are also listed.

### 2.2 Initial and Comprehensive Ligand Data Sets

First, the initial ligand data set, also called the training set, included 28 complexes: 18 full agonists and 10 partial agonists, curated from the previous collection of PPARγ-ligand complexes in the PDB. (Table S1) Full agonists were defined as having a maximum activation efficacy (Emax) ≥90%, whereas partial agonists had an Emax ≤ 45%. These empirical thresholds were chosen to maximize the signal-to-noise ratio for statistical analysis. The ≥90% cutoff accounts for typical experimental variability while ensuring undisputed full agonism, whereas the ≤45% boundary ensures a sufficient sample size of partial agonists. A buffer zone between these thresholds explicitly excluded intermediate-efficacy ligands to prevent classification ambiguity and mitigate experimental uncertainty. Here, we compiled a polarized, focused subset to test the essential parameters of the Ligand Drift Rate (LDR) Descriptor and to develop a robust computational framework for molecular docking parameter settings.

Second, to develop a complete PPARγ ligand data set, called the validation set, we extracted 4698 experimental records from the ChEMBL database categorized under human PPARγ activity. These records included various efficacy measurements from published studies (such as Emax, %max, intrinsic activity, and fold activation). After removing nonquantitative and irrelevant data (like missing values, nonpercentage measurements, or concentration-based readouts), we retained 1816 entries with well-documented maximal activation efficacy values. Additionally, to address inconsistencies caused by different reference standards (such as varying full agonists used as controls), we normalized all efficacy values to rosiglitazone (Emax = 100%), facilitating cross-study comparisons where multiple standards were benchmarked. This normalization resulted in 1561 molecule ligands (Table S2) with Emax values scaled to Rosiglitazone. Furthermore, we obtained the 2D structures of these compounds in SDF format through a batch search using their ChEMBL IDs, ensuring chemical consistency for future analyses. Using these polarized thresholds, compounds were again classified as full agonists (Emax ≥ 90%) or partial agonists (Emax ≤ 45%). Ultimately, an extended ligand data set of 1,079 ligands (including 393 full agonists and 686 partial agonists) was compiled from the ChEMBL database, offering a broad chemical and pharmacological range of all current PPARγ ligands. Unlike the initial PDB-derived set, which was based on crystallographic snapshots of bound ligands, this ChEMBL data set included compounds without resolved cocrystal structures, encompassing noncovalent binders and diverse scaffolds (such as carboxylic acids and sulfonamides). Therefore, we established a comprehensive data set to validate the LDR Descriptor.

Third, all the curated compounds in either the initial or complete ligand data set were geometrically optimized within the Discovery Studio (DS) 2019 environment (BIOVIA, Dassault Systèmes, San Diego, CA, USA) using the MMFF force field^22^ until the structure’s energy reached a RMSD gradient of 0.05 kcal mol^–1^ Å^–1^. After geometric optimization, partial charges for all compounds were explicitly calculated and assigned using the MMFF force field, and the resulting structures were automatically saved in SDF format.

### 2.3 Molecular Docking Sampling

Molecular docking is a structure-based computational method that, as originally designed, predicts the preferred binding orientation and affinity of a small-molecule ligand within the binding site of a target protein. However, we use molecular docking as a sampling technique to evaluate docking poses that escape from the primary binding site, as described in the two-state model. The docking algorithm, LigandFit,^23^ was preliminarily assessed using the 6ENQ-BJB binding pocket to identify the number of retained poses after molecular docking sampling. Then, four docking algorithms (CDOCKER, LibDock, Flexible Docking, and GOLD)^24–28^ were used to identify the most effective molecular docking strategy for discriminating between full and partial agonists using the LDR descriptor in the training set. Based on their discriminatory capabilities, all subsequent molecular docking analyses were performed using the GOLD (Genetic Optimization for Ligand Docking) program. GOLD utilizes a genetic algorithm to dock flexible ligands into the receptor’s binding site. Before molecular docking, the binding site was defined using the edit binding site tool, specifying a spherical region centered on the ligand in each crystal complex. The binding space expands until it reaches surrounding residues, a default setting. Protein structures were preprocessed by removing crystallographic water molecules and nonessential ions and adding hydrogens to complete valence shells. Ligands were imported in SDF format with all rotatable bonds preserved to allow conformational flexibility during docking. Molecular docking calculations were initially performed using the GOLD default genetic algorithm settings to ensure high predictive accuracy. Each ligand was docked 100 times (Number of Dockings = 100) to improve a comprehensive collection of predicted binding poses. We used genetic algorithm settings with population size = 100, generations = 100, crossover rate = 95%, and mutation rate = 5% to generate 100 conformations per ligand, ranked by score. The GoldScore scoring function evaluated binding poses, considering hydrogen bonding, van der Waals interactions, and ligand torsional strain. The early termination option was not enabled.

The diversity option in GOLD controls the level of conformational diversity by setting a minimum root-mean-square deviation (RMSD) threshold to guarantee sufficient structural differences between generated docking conformations. The diverse solution option can be enabled to ensure pose diversity with an RMSD threshold of 0.5 Å and a cluster size of 1, as defined. Then, the program removes candidate conformations that are similar to already accepted conformations, creating a more varied set of docking poses. This mechanism helps prevent conformational clustering, ensuring that the final docking results more thoroughly explore possible ligand binding modes within the binding pocket, thereby enhancing docking quality and the accuracy of biological activity predictions.

The Flexible option in GOLD is another control that offers receptor flexibility. In GOLD, this is achieved by allowing flexible side chains within the binding pocket, enabling key residues to adopt different conformations during docking and better accommodating ligand binding through induced-fit mechanisms. During molecular docking, flipping situations can involve ring corners, amide bonds, and all planar R-NR1R2 bonds. Protonated carboxylic acid and all rotatable bonds can rotate freely. This flexibility setting for residues in the binding site was used in the secondary run of both the initial and comprehensive data sets.

### 2.4 Definition of Ligand Drift Rate

The ligand drift rate quantifies the positions of receptor ligands. After 100 molecular docking iterations, the top 20 poses with high docking scores are used to calculate the ligand drift rate by counting the number of poses that drift. The drift rate is defined as

Ligand Drift Rate(%)=(N/20)×100

where *N* represents the number of the top 20 poses that fail to maintain close proximity to all three key AF-2 residues (His323, His449, and Tyr473). For example, a ligand drift rate of 25% means that 5 poses have drifted away from these key anchoring points. Here, the postdocking analysis was performed using a custom Perl script within Discovery Studio to automate the extraction of key residues, rank the top 20 ligand poses by Goldscore Fitness, and systematically quantify spatial displacement.

To evaluate the two-state model, spatial proximity thresholds (hereafter referred to as hydrogen-bond displacement, or HBD, thresholds) were set to 6, 8, 10, and 12 Å. Although the selection of interacting atoms follows IUPAC conventions for potential hydrogen-bond donors/acceptors, the extended distances are used strictly as a spatial metric to quantify displacement from the canonical hydrogen-bond anchoring sites of the full agonist state. The HBD threshold defines the boundary between the two states, which in turn determines ligand drift. In simple terms, docking poses where the distance to these key residues exceeds the defined HBD threshold are classified as drifted.

### 2.5 Two-Sample *t*-Test Analysis

The Ligand Drift Rate (LDR) descriptor was used to characterize the different sampling behaviors between PPARγ full and partial agonists during molecular docking simulations. LDR quantifies the dynamic positional changes of ligands across various docking conditions and hydrogen-bond displacement (HBD) cutoff thresholds. Violin plots were created using GraphPad Prism 8 (GraphPad Software, www.graphpad.com) to show the distribution of LDR values for each agonist class. To see if full and partial agonists showed statistically significant differences in LDR, two-sample independent *t*-tests were conducted. The analysis included multiple HBD cutoff thresholds to systematically evaluate how changing hydrogen-bonding criteria affects the separation between agonist classes. For each HBD threshold, LDR values were compared between full and partial agonist groups assuming: (1) data were drawn from normally distributed populations (Gaussian distribution), (2) samples were randomly and independently selected from each population, and (3) variances were roughly equal between the two groups (homoscedasticity). All tests were two-tailed with a significance level of α = 0.05. This multithreshold approach provided a systematic way to understand how hydrogen-bonding stringency influences the LDR descriptor’s ability to distinguish between full and partial agonists based on differences in their molecular dynamics and binding behavior.

## 3 Results and Discussion

### 3.1 Two-State Model and Molecular Docking Sampling

The maximum transcriptional activation efficiency of nuclear receptors is determined by the degree of ligand occupancy at the orthosteric binding site. At saturation, all available receptor binding sites are occupied by ligand molecules in a thermodynamically favorable manner. Under these conditions, ligand occupancy at the orthosteric site follows a Boltzmann distribution, where the population of bound states is proportional to the relative free energy differences between conformational states.

$$P_{1}=\frac{e^{-G_{1}/k_{B}T}}{\underset{i}{\sum }e^{-G_{i}/k_{B}T}}$$

where *P*_1_ denotes the probability of ligand binding at the state 1 (the primary orthosteric binding site), and *i* denotes all possible ligand binding states, including the situation, *i* = 1. *G*_*i*_ represents the Gibbs free energy at *i* state, *k*_B_ is Boltzmann’s constant, and *T* is the absolute temperature. Notice that the occupation of ligands at the primary binding site with key hydrogen bonding interactions determines the receptor’s ability to activate transcription.

To adequately capture the Boltzmann distribution on a multifarious energy landscape of the conformational ensemble that governs ligand-protein binding, this study employs molecular docking sampling to directly estimate binding probabilities at the primary orthosteric site and to evaluate ligand occupancy. It can be viewed as a two-state binding model within the NR system (Figure 1). Based on the key AF-2 interaction between NR and its ligand, the primary orthosteric binding site of NR can be defined before molecular docking sampling by its structural features and canonical ligand interactions. However, many alternative binding sites remain unknown in advance due to experimental limitations and the time-consuming nature of simulations. Thus, all other binding sites are grouped into “the other state”; the actual binding situation may involve a specific conformational change before the ligand binds. Additionally, since agonist activity requires stabilization of helix 12 through hydrogen bonding with the AF-2 residues as mentioned earlier, we established the following classification criterion: ligands positioned near these residues but lacking the necessary hydrogen bonds are classified as occupying the alternative binding sites, the second state. This occupancy-based sampling scheme allows prediction of population-level nuclear receptor (NR) activation by linking transient active ligand binding to fractional transcriptional outcomes. Accordingly, the hydrogen-bond displacement (HBD) threshold defines the boundary between the primary and the other states within the molecular binding domain, serving as a critical criterion. Taken together, the primary state is conclusively defined as a ligand–receptor binding configuration where the ligand occupies the primary orthosteric binding site and forms hydrogen bonds with key AF-2 residues (Tyr473, His449, His323) within the predefined hydrogen-bond displacement threshold, thereby stabilizing helix 12 for coactivator recruitment and transcriptional activation. The fractional population of partial agonists shifts to the other state, i.e., the alternative binding sites, at saturating concentrations, resulting in submaximal receptor activation and reduced transcriptional efficacy compared with full agonists. Leveraging this characteristic of the two-state model, a molecular descriptor is constructed via molecular docking sampling techniques. This descriptor can be used to distinguish between full and partial agonists.

**Figure 1:**
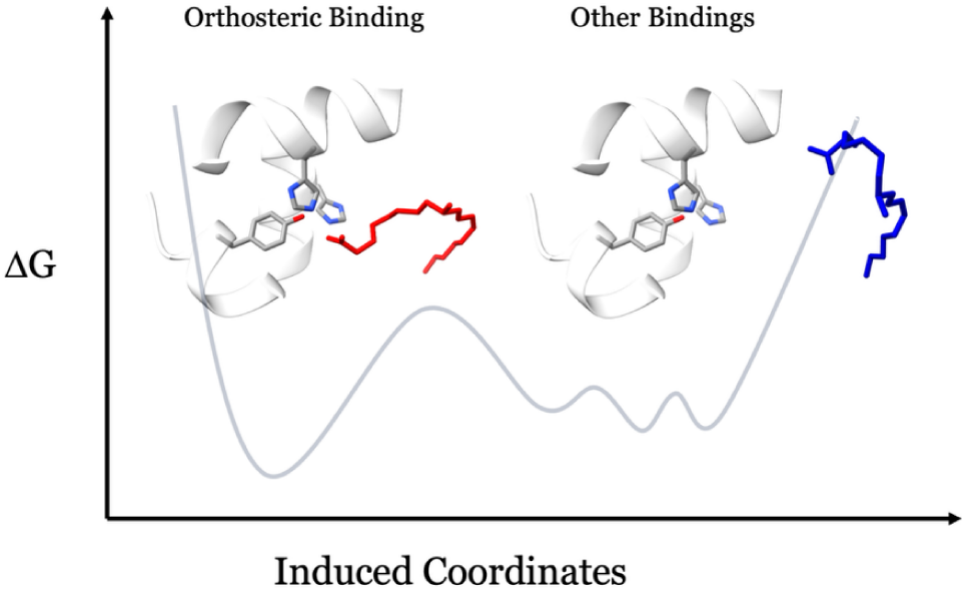
Schematic energy diagram for the two-state binding model of nuclear receptor ligand interactions, distinguishing between full and partial agonists. The potential energy diagram shows the conformational energy landscape along the interaction coordinates. Two binding scenarios are depicted: the left structure illustrates a ligand (red) bound to the primary orthosteric site with proper hydrogen bonding to AF-2 residues (stabilizing helix 12), while the right structure shows a ligand (blue) occupying an alternative binding site without essential hydrogen bonds. Full agonists mainly occupy the primary state (left energy well) with stable AF-2 interactions, whereas partial agonists switch between the primary orthosteric site and other binding configurations (right energy well), resulting in submaximal receptor activation. The hydrogen-bond displacement (HBD) threshold to key AF-2 residues (Tyr473, His449, His323) is the critical criterion for differentiating these two binding states. Gray helical ribbons represent the protein’s secondary structures. The gray wave line sketches the energy landscape along the possible induced-fit interaction coordinates.

### 3.2 Retaining 20 Top Docking Poses for Better LDR’s Differentiation

To optimize molecular docking parameters for distinguishing between 18 full agonists and 10 partial agonists, we examined the number of retained poses within the binding pocket in 6ENQ-BJB. First, we tested 100, 20, and 10 retained docking poses at hydrogen bond thresholds of 6, 8, 10, and 12 Å using the 6ENQ-BJB binding site for ligand drift rate calculations. All final retained ligand poses were selected from the final 100 docking poses ranked by the PMF scoring function. Under the condition of 100 retained poses, statistical tests (Figure 2A) did not reach significance (the best p-value was 0.099 at 12 Å), with none below 0.05. In contrast (Figure 2B), 20 retained poses achieved statistical significance at both 10 Å and 12 Å HBD thresholds (*p*-values of 0.013 and 0.00043, respectively). More observations (Figure 2C) showed that 10 retained poses yielded similar results under the same conditions (p-values of 0.022 and 0.00044). This finding revealed that partial agonists consistently exhibited higher ligand drift rates than full agonists, providing initial support for the core hypothesis that hydrogen bonds formed by partial agonists with key amino acids are more prone to drift. Considering both statistical power and experimental efficiency, 20 retained docking poses were selected as the optimal parameter setting for effectively distinguishing between the two agonist types, establishing a reliable foundation for subsequent research.

**Figure 2:**
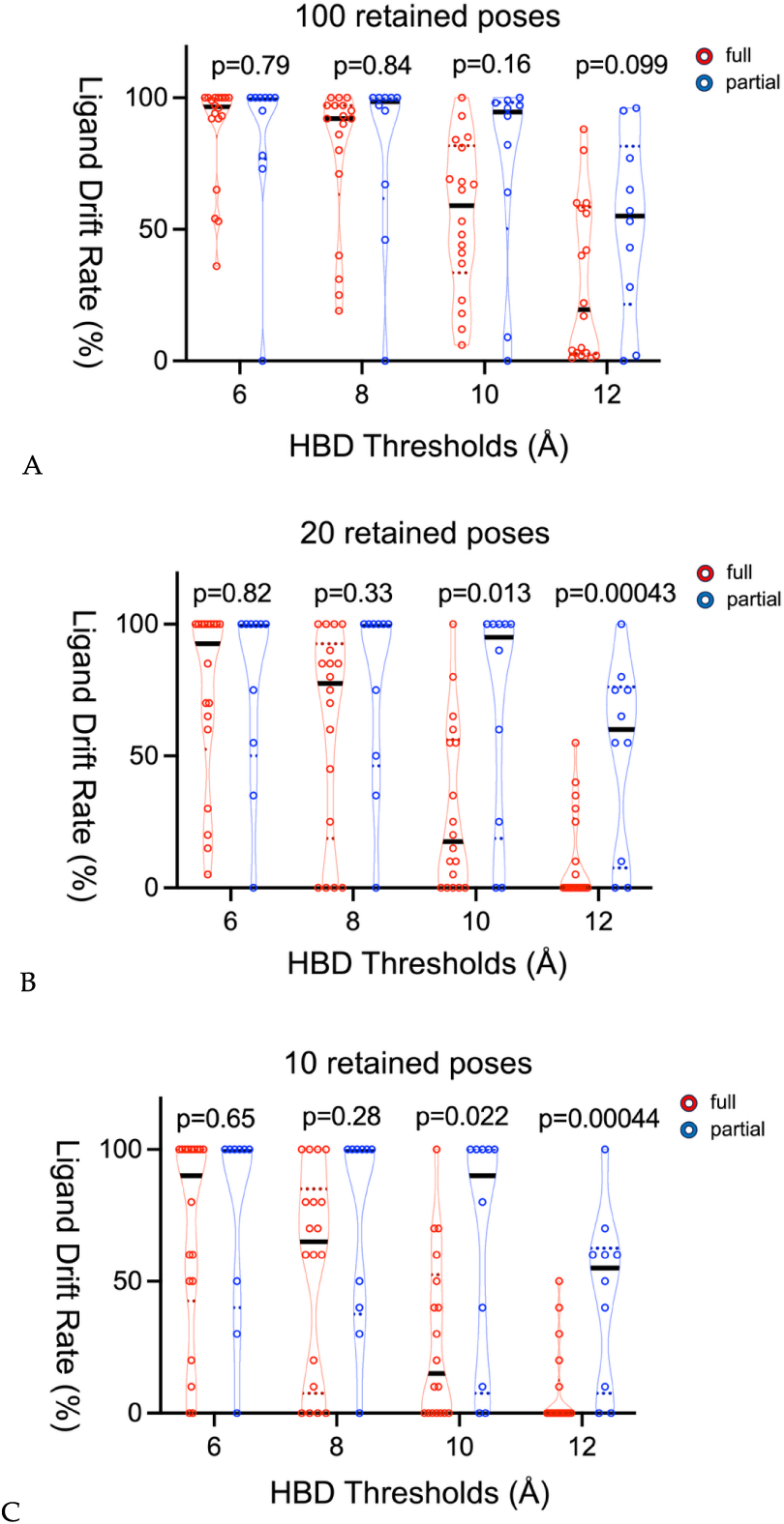
Comparison of ligand drift rates between full and partial agonists under different docking pose retention conditions.

Violin plots illustrating the distribution of ligand drift rates (%) for full agonists (red) and partial agonists (blue) across different hydrogen-bond displacement thresholds (HBD) (6 Å, 8 Å, 10 Å, and 12 Å). (A) Results with 100 retained docking poses show no statistically significant difference (*p* > 0.05 at all HBD thresholds). (B) Results with 20 retained docking poses reveal statistically significant differences at 10 Å (*p* = 0.013) and 12 Å (*p* = 0.00043) HBD thresholds. (C) Results with 10 retained docking poses demonstrate similar significance at 10 Å (*p* = 0.022) and 12 Å (*p* = 0.00044) thresholds. Individual data points are overlaid on violin plots, with horizontal black bars indicating medians. Partial agonists consistently show higher drift rates than full agonists at longer HBD thresholds, with 20 retained poses identified as the best parameter for distinguishing between the two types. *P*-values are calculated and shown.

### 3.3 Genetic Algorithm Has Reasonable LDR’s Discriminatory Power

To identify the optimal molecular docking strategy for distinguishing between full and partial agonists in the training set, four algorithms (CDOCKER, LibDock, Flexible Docking, and GOLD) were evaluated using the 6ENQ-BJB binding pocket. Each algorithm generated 20 top-ranked poses per ligand, and drift rates were calculated across multiple hydrogen-bond displacement (HBD) thresholds (6, 8, 10, and 12 Å). Results (Figure 3) showed that CDOCKER, LibDock, and Flexible Docking produced similar drift rate profiles, with mean drift rates decreasing as hydrogen-bond displacement increased—an expected trend. However, all three methods had *p*-values above 0.4, possibly indicating limited ability to distinguish between agonist types. We also observed some reverse drift rates (CDOCKER at 10 Å, and LibDock at 6 Å), where full agonists unexpectedly showed higher average drift rates than partial agonists. This challenges the reliability of certain docking methods for this sampling strategy aimed at capturing the Boltzmann distribution, including LigandFit, which also exhibited opposite drift rates in the validation set (data not shown). Only GOLD achieved the lowest *p*-value (*p* = 0.26) at a 10 Å HBD threshold without reverse drift rates, demonstrating superior power among the docking strategies. The genetic algorithm in GOLD was chosen for further validation. Then, continuous parameter optimization of molecular docking sampling was performed across five receptor pockets (3B1M-KRC, 6ENQ-BJB, 6ICJ-A0L, 6ILQ-AE0, and 6T6B-MLQ) to identify the optimal binding pocket for molecular docking sampling.

**Figure 3:**
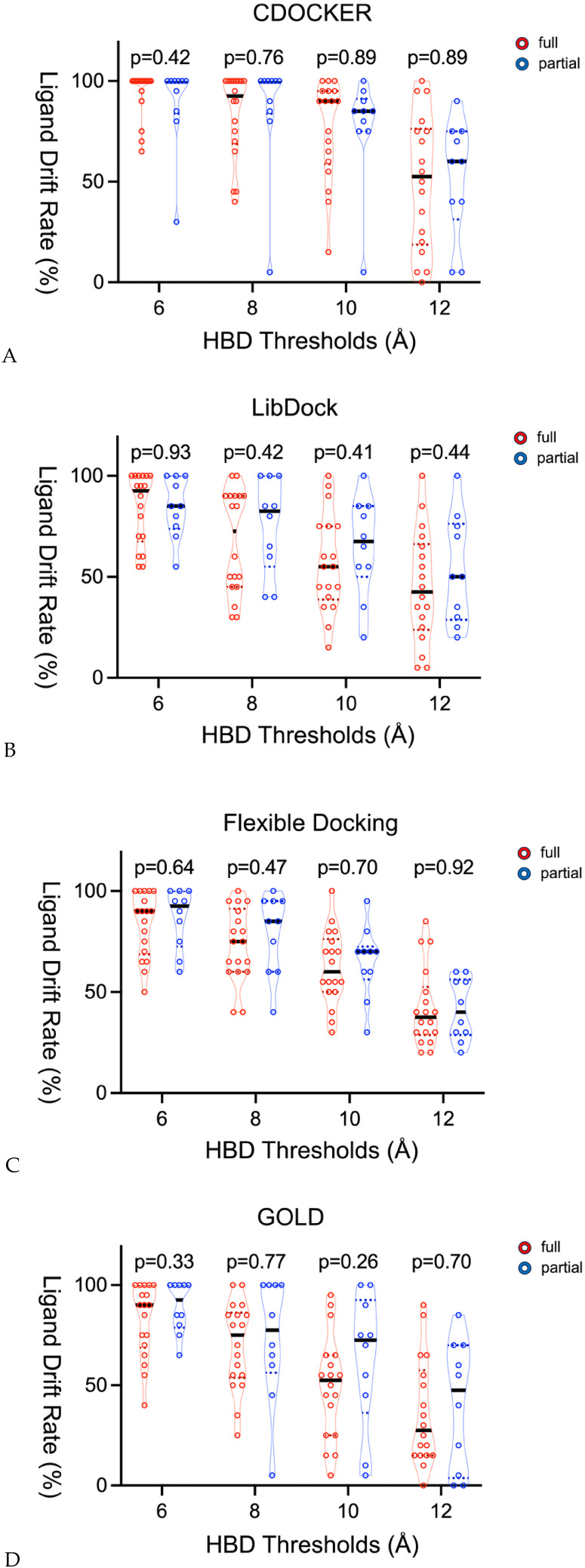
Comparative evaluation of ligand drift rates across four molecular docking algorithms for discriminating full and partial agonists. Ligand drift rates were calculated for full agonists (red) and partial agonists (blue) using the 6ENQ-BJB binding pocket at HBD thresholds of 6, 8, 10, and 12 Å. Each algorithm generated 20 top-ranked poses per ligand from the training set. (A) CDOCKER, (B) LibDock, (C) Flexible Docking, and (D) GOLD. Violin plots display the distribution of LDRs, with individual data points overlaid; horizontal black bars indicate the median. P-values above each threshold indicate statistical differences between agonist classes by a two-sample *t*-test. CDOCKER, LibDock, and Flexible Docking showed *p*-values >0.4 (very poor differentiation) with occasional reverse drift patterns. At the same time, GOLD demonstrated the best discriminatory power with the lowest *p*-value (*p* = 0.26 at 10 Å) and consistent ligand drift rate patterns, where partial agonists showed higher ligand drift rates than full agonists across all thresholds.

### 3.4 3B1M-KRC Binding Pocket Shows Excellent LDR’s Discrimination

Following the selection of GOLD’s genetic algorithm based on its discrimination ability, we systematically examined all five PPARγ binding pockets (3B1M-KRC, 6ENQ-BJB, 6ICJ-A0L, 6ILQ-AE0, and 6T6B-MLQ) to find the most effective receptor-binding pocket for distinguishing between full and partial agonists. These pockets were previously chosen from 356 PPARγ-ligand complexes in the Protein Data Bank using strict criteria: high-resolution structures (≤2.5 Å), the largest binding pocket volumes (top 10%), and conformations with full agonists that support the lock-and-key binding model. All selected structures preserved the orthosteric AF-2 activation conformation, with the correct spatial arrangement of key residues His323, His449, and Tyr473.

Molecular docking sampling with GOLD’s default settings revealed pocket-dependent performance patterns that differed from those observed in the HBD threshold-responsive analysis. As shown in Figure 4, the violin plot analysis clearly identified the 3B1M-KRC binding pocket as the most discriminating among the five, achieving statistically significant separation between full and partial agonists at the 10 Å hydrogen bond threshold (*p* = 0.043, α = 0.05). In contrast, the 6ENQ-BJB pocket showed considerable overlap in ligand drift rates between full and partial agonists across all tested HBD thresholds, resulting in no statistically significant difference, consistent with our previous four-algorithm comparison. Similarly, both 6ICJ-A0L and 6T6B-MLQ pockets failed to produce meaningful separation between agonist classes. The 6ILQ-AE0 pocket showed slight discriminatory potential at the 8 Å threshold, though the separation remained statistically nonsignificant (*p* = 0.080, α = 0.05). These findings identify the 3B1M-KRC crystal snapshot as the optimal binding pocket for GOLD-based agonist classification, indicating that specific receptor conformations and binding-site architecture are key factors influencing algorithm performance. This supports our hypothesis of pocket-algorithm compatibility and provides a solid basis for subsequent drift-rate analyses. We suggest that the superior discriminatory ability of the 3B1M-KRC pocket results from its structural conformation, which more accurately captures the distinct active and inactive receptor states inherent to the two-state receptor activation model. This enables GOLD to differentiate the binding energetics and interaction patterns associated with full versus partial agonists.

**Figure 4:**
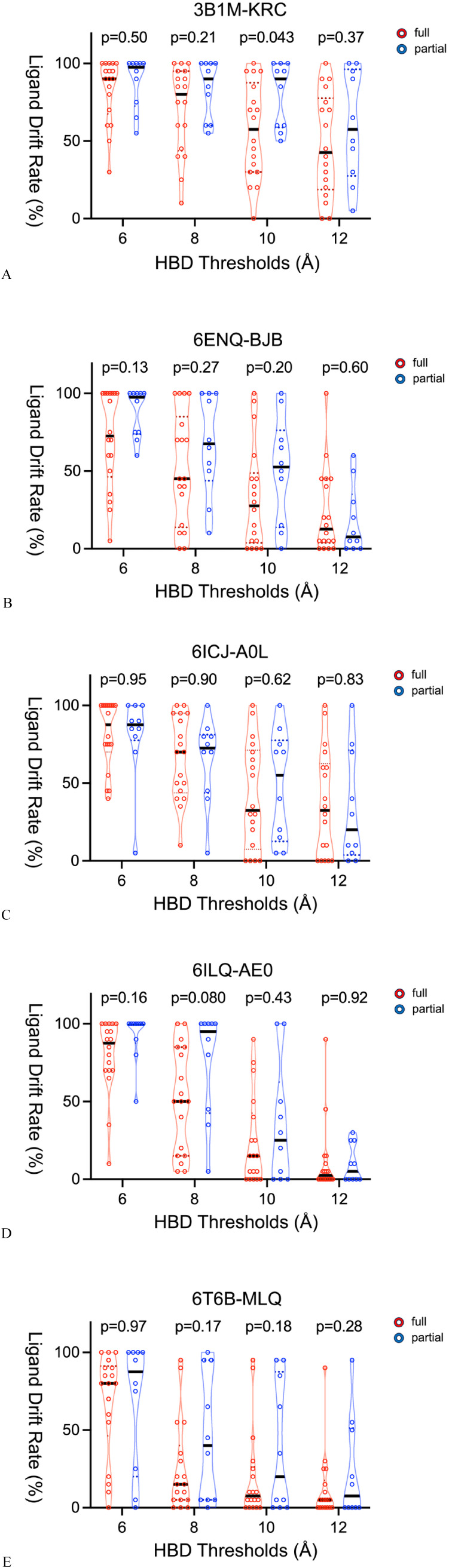
Systematic evaluation of five PPARγ binding pockets using GOLD’s genetic algorithm to identify the best receptor structures for differentiating full and partial agonists.

Violin plots illustrating the distribution of drift rates (%) for full agonists (red) and partial agonists (blue) across four hydrogen-bond displacement thresholds (6 Å, 8 Å, 10 Å, and 12 Å) using 20 retained docking poses per ligand in the training set. (A) The 3B1M-KRC binding pocket shows exceptional discriminatory ability, achieving a statistically significant separation between full and partial agonists at 10 Å (*p* = 0.080, α = 0.05), with clearly distinct distribution patterns between the two classes. (B) The 6ENQ-BJB binding pocket displays considerable overlap between full and partial agonist drift rates across all HBD thresholds, with no statistical significance (*p* > 0.05), consistent with previous four-algorithm comparison results. (C) The 6ICJ-A0L binding pocket fails to produce meaningful separation between agonist classes at any tested threshold (*p* > 0.05). (D) The 6ILQ-AE0 binding pocket shows marginal discriminatory potential at 8 Å (*p* = 0.080) but remains statistically nonsignificant (*p* > 0.05). (E) The 6T6B-MLQ binding pocket demonstrates no discriminatory power across all HBD thresholds (*p* > 0.05). Individual data points are overlaid on violin plots, with horizontal black bars indicating median values. These results identified the 3B1M-KRC crystal snapshot as the optimal binding pocket for GOLD-based agonist classification, highlighting that specific receptor conformational features and binding site architecture play essential roles in algorithm performance and supporting the pocket-algorithm compatibility hypothesis.

### 3.5 Pose Diversity and Receptor Flexibility Enhance LDR Discrimination

To systematically evaluate factors affecting the discriminatory power of the LDR, we examined three progressive docking parameter configurations for PPARγ full and partial agonists binding to the 3B1M-KRC pocket (Figure 5). Under default GOLD docking parameters, full agonists showed significantly lower drift rates (median ∼60%) compared to partial agonists (median ∼85%), with statistical significance at *p* = 0.043. This initial finding suggested that LDR captures fundamental differences in binding behavior between agonist classes, consistent with the idea that full agonists form more stable interactions that resist conformational drift during molecular saturation. Next, applying a pose diversity setting with a 0.5 Å RMSD threshold greatly improved discrimination (*p* = 0.022), indicating that thorough sampling of ligand conformational space is crucial for LDR accuracy. This improvement aligns with established molecular docking principles, suggesting that generating diverse poses better reflects the true energy landscape of the two-state model. Finally, combining conformational diversity constraints with receptor flexibility parameters achieved the best discrimination (p = 0.014), further enhancing the results seen with default settings. This stepwise enhancement demonstrates that considering both ligand positional sampling and receptor flexibility is vital for LDR to accurately differentiate the binding behaviors of various potential ligand agonists. The findings support the induced-fit model of ligand binding, where receptor flexibility plays a key role in determining the binding outcome. Full agonists consistently showed lower drift rates, indicating they induce more stable receptor-active conformations that stay close to the AF-2 coactivator-binding surface, the primary orthosteric site. In contrast, partial agonists exhibited higher LDR, suggesting they tend to occupy alternative conformations where the ligand-binding pocket is shifted away from the AF-2 region, leading to less helix 12 stabilization and reduced coactivator recruitment. These results emphasize that the LDR Descriptor’s ability to distinguish pharmacological classes depends critically on capturing the dynamic protein–ligand recognition landscape through appropriate molecular sampling strategies. The inclusion of pose diversity and receptor flexibility further improved the performance of the GOLD algorithm in agonist classification, especially for the 3B1M-KRC binding pocket. This solidified its position as the best receptor structure and enhanced its statistical ability to distinguish.

**Figure 5:**
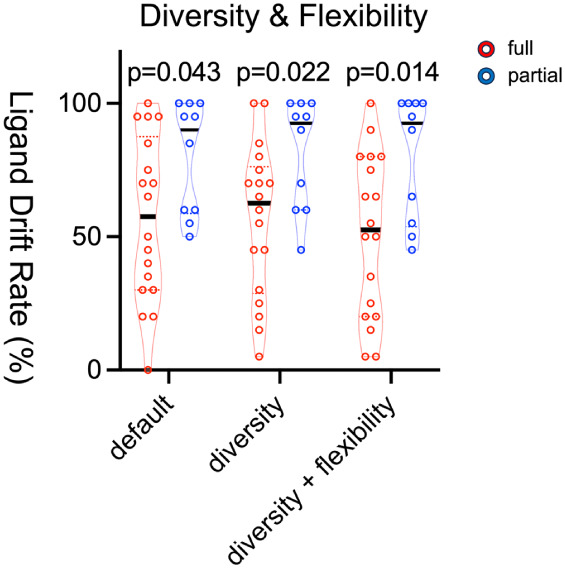
Pose diversity and receptor flexibility on LDR discrimination.

Violin plots compare LDR (%) for PPARγ full agonists (red) and partial agonists (blue) using the 3B1M-KRC binding pocket across three progressively different docking parameter setups: default settings, pose diversity (0.5 Å RMSD threshold), and combined diversity with receptor flexibility. Each circle represents an individual ligand, with horizontal black bars indicating median values and dotted lines showing quartiles. The statistical significance between full and partial agonists improves across configurations (*p* = 0.043, 0.022, and 0.014, respectively). Full agonists consistently show lower drift rates (<56.4%), suggesting most bind near the AF-2 coactivator-binding surface, while partial agonists have higher drift rates (>80.0%), indicating binding at alternative sites with less helix 12 activation. The increasing discrimination emphasizes that considering pose diversity and receptor flexibility is key for the LDR descriptor to effectively distinguish agonist classes based on binding behavior and receptor conformations.

### 3.6 Large-Scale ChEMBL Data Set Confirms the Validity of LDR Descriptors

After establishing optimal parameters through systematic training set tuning (3B1M-KRC binding pocket, GOLD genetic algorithm, 10 Å HBD threshold, with pose diversity and receptor flexibility enabled), we evaluated the LDR descriptor on a large data set of 1,079 PPARγ ligands from ChEMBL. This data set included 393 full agonists (Emax ≥ 90%) and 686 partial agonists (Emax ≤ 45%). It represented a significant expansion beyond the training set’s crystallographic ligands, covering diverse chemical scaffolds without resolved cocrystal structures, including more carboxylic acids and sulfonamides with various structural components. As shown in Figure 6, full agonists consistently showed significantly lower drift rates than partial agonists when combining pose diversity with a 0.5 Å RMSD threshold and receptor flexibility. Notably, this large-scale validation yielded the strongest statistical significance (*p* = 0.00043) compared to the training set (*p* = 0.041), supporting the two-state binding model.

**Figure 6:**
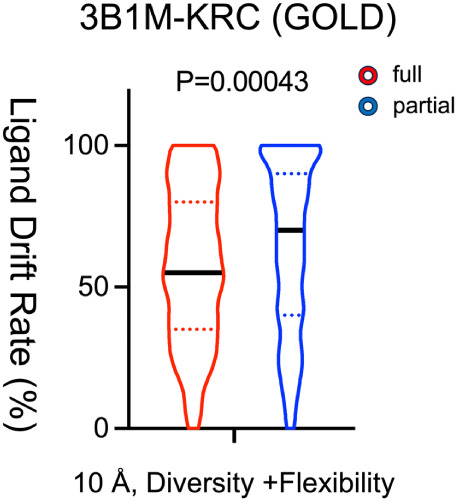
Validation of LDR discrimination using a comprehensive ChEMBL data set of 1,079 PPARγ ligands.

These results demonstrate that the LDR descriptor effectively captures key differences in binding behavior between agonist classes by systematically exploring ligand docking pose space. Mechanistically, this robust performance in distinguishing full from partial agonists stems from the distinct thermodynamic landscapes these ligands induce. While classical full agonists firmly anchor key residues on the AF-2 surface, locking the receptor into a deep, narrow energy minimum, partial agonists allow the receptor to occupy a broader conformational energy well. The “drifted” poses predominantly observed in partial agonists thus reflect the ligand’s propensity to explore multiple local energy minima. This computational observation aligns well with the dynamic helix 12 conformations and slow chemical exchanges previously confirmed by physical NMR experiments. Therefore, the heuristic spatial sampling provided by our optimized molecular docking protocol serves as a highly effective, practical proxy for the underlying Boltzmann distribution of these microstates. By providing a clear mechanistic bridge between computational ligand positioning and in vitro pharmacological efficacy, this extensive validation across diverse chemical scaffolds ultimately confirms LDR as a reliable computational descriptor for agonist classification in structure–activity relationship studies and prospective ligand design.

Violin plots comparing ligand drift rate (%) distributions for full agonists (red, *n* = 393, Emax ≥ 90%) and partial agonists (blue, *n* = 686, Emax ≤ 45%) using the optimized 3B1M-KRC binding pocket with GOLD’s genetic algorithm at a 10 Å hydrogen-bond displacement threshold. The progressive docking configurations are shown: default GOLD parameters, pose diversity enabled with 0.5 Å RMSD threshold, and combined pose diversity with receptor flexibility. No circle represents due to too many ones. The horizontal black bars indicate median values, and the dotted lines show quartile boundaries. Statistical significance between full and partial agonists shows a distinguishable capability (*p* = 0.00043). This superior predictive performance of the 3B1M-KRC template may be driven by its distinct structural attributes, as detailed in Table 1: maximum efficacy (Emax), X-ray resolution, and binding pocket volume. First, while 3B1M-KRC presents the active, full-agonist-bound pocket conformation required for baseline docking, it is uniquely cocrystallized with a partial agonist (Emax = 37%), unlike the other templates bound by highly potent full agonists (Emax ≥ 93%). This is strongly supported by the original crystallographic study of 3B1M, which revealed that the cocrystallized partial agonist (KRC) does not directly interact with Helix 12, resulting in a less rigidly stabilized active conformation.^29^ This unique pocket environment maintains the active AF-2 architecture without the extreme structural rigidity induced by true full agonists, thereby allowing partial agonists to capture dynamic drift behavior without artificial steric suppression. Second, its exceptionally high X-ray resolution (1.60 Å) minimizes coordinate noise in critical anchoring residues (His323, His449, and Tyr473). Finally, its well-balanced volume (525.875 Å^3^) provides sufficient spatial flexibility for conformational exploration while avoiding unphysical conformational sampling during docking. Consequently, full agonists consistently exhibit lower drift rates (median = 57.46%), indicating stable binding at the primary orthosteric site with preserved AF-2 interactions (reflecting a deep energy minimum), while partial agonists show higher drift rates (median = 63.9%), reflecting their tendency to occupy alternative binding subpockets within a broader energy well. The progressive enhancement in discrimination once again confirms the mechanistic importance of MODO sampling and receptor flexibility in reflecting the true landscape of the two-state model. This large-scale validation across diverse chemical scaffolds establishes LDR as a useful computational metric for agonist classification in structure–activity relationship studies and prospective ligand design.

## 4 Conclusion

This study introduces the development and validation of the Ligand Drift Rate (LDR) descriptor, a novel computational metric that quantitatively differentiates partial from full agonists at PPARγ using a Boltzmann-distribution-based two-state model. By explicitly modeling ligand positional instability, or ligand drift, the LDR framework addresses a significant need in computational chemistry and structure-based drug design. Systematic parameter optimization established a reliable protocol for calculating the LDR, utilizing GOLD’s genetic algorithm, the 3B1M-KRC receptor structure, and optimized settings (top 20 out of 100 final docking poses, 10 Å Hydrogen-Bond Displacement (HBD), 0.5 Å RMSD pose diversity, flexible side chains). The remarkable two-orders-of-magnitude increase in statistical significance from the training set (*p* = 0.041) to the large-scale ChEMBL validation set (*p* = 0.00043; 393 full agonists, 686 partial agonists) confirms that our dynamic sampling robustly reflects pharmacologically relevant binding behavior. The consistent trend of higher drift rates for partial agonists than for full agonists demonstrates that LDR effectively captures essential mechanisms underlying ligand-binding dynamics. Mechanistically, this quantified positional drift reflects the ligand’s propensity to explore a broader conformational energy well, which directly correlates with the transient occupation of alternative binding subpockets, reduced proximity to critical AF-2 residues, and the characteristic destabilization of Helix 12. Conversely, the lower drift rates of full agonists represent stable binding that firmly locks the receptor into a deep, narrow energy minimum.

The LDR framework offers immediate practical value for computational drug discovery programs targeting PPARγ and other nuclear receptors. Pharmacologically, distinguishing full agonists is critical; partial agonists can maintain therapeutic efficacy (e.g., insulin sensitization) while avoiding severe side effects, such as elevated cardiovascular risk. By leveraging the computational efficiency of molecular docking (MODO) sampling, LDR calculations enable large-scale virtual screening and support the early identification of selective nuclear receptor modulators. While LDR already demonstrates exceptional discriminative power on its own, incorporating it alongside existing descriptors into multiparametric or machine-learning models can further improve predictive accuracy. Furthermore, this methodological framework can be readily adapted to other nuclear receptor family members and allosteric proteins where graded activation is a therapeutically relevant strategy. In summary, the LDR descriptor is a significant advance in cheminformatics, providing the first quantitative metric that links computational ligand spatial distributions to in vitro maximum pharmacological efficacy. By explicitly accounting for the dynamics of partial agonism through efficient Boltzmann statistical mechanics, this work underscores the broader potential of dynamics-aware molecular descriptors in targeted therapeutic design.

## Supplementary Material



## Data Availability

The data sets, molecular structure files (.sdf) used in this study, the illustrated Table S1 and S2 for training and testing sets (.docx), the Perl script used in the Discovery Studio environment for statistical evaluation of pose hydrogen-bond diversity (.pl), and the ligand drift pose count for validation set (.csv) are available at: https://github.com/ytlin168/LDR_Descriptor/

## Notes

Table S1 lists the training set of PPARγ-ligand complexes used in the Ligand Drift Rate analysis. Table S2 lists the validation set of PPARγ ligands curated from the ChEMBL Database. Both tables report each ligand’s maximum efficacy as a percentage relative to Rosiglitazone. (PDF)
