# Supplementary material for: Ligand Drift Rate Descriptor in Receptor Pocket: Molecular Docking Sampling for Differentiating Partial Agonists in Nuclear Receptors
Source: J Chem Inf Model. 2026 Jul 23;66(15):9089–98. doi: 10.1021/acs.jcim.6c00669 (PMC13471412; doi:10.1021/acs.jcim.6c00669)
Supplement: Supplementary file 1 [file ci6c00669_si_001.pdf]

Supporting Information

Ligand Drift Rate Descriptor in Receptor Pocket: Molecular Docking Sampling for Differentiating Partial Agonists in Nuclear Receptors

Ying-Ting Lin [1,2 *],and Yung-Ting Shih [1]

1. Department of Biotechnology, College of Life Science, Kaohsiung Medical University, Kaohsiung 807, Taiwan
2. Drug Development & Value Creation Research Center, Kaohsiung Medical University, Kaohsiung 807, Taiwan

*Corresponding author: Ying-Ting Lin (ytlin@kmu.edu.tw)

Contents

Supplementary Table S1. Training Set of PPARγ-Ligand Complexes Used for Ligand Drift Rate Analysis, Including the Ligands' Maximum Efficacy.… S3-S4

Supplementary Table S2. Validation Set of PPARγ Ligands Curated from the ChEMBL Database, Including the Ligands' Maximum Efficacy. …...……S5-S49

Table S1. Training Set of PPARγ-Ligand Complexes Used for Ligand Drift Rate Analysis

Curated collection of 28 PPARγ-ligand crystal structures from the Protein Data Bank (PDB) comprising 18 full agonists and 10 partial agonists. The table includes PDB identifiers, ligand names/codes, and maximum efficacy for each complex ligand used in the initial development and validation of the ligand drift rate descriptor.

Molecule PDB ID	Relative to Rosiglitazone
2I4J_DRJ	116%
2Q8S_L92	102%
2Q59_240	97%
3BC5_ZAA	138%
3CDS_GRR	116%
3IA6_UNT	112%
3v9y_24L	105%
5U5L_7VA	126%
6ENQ_BJB	97%
6ILQ_AE0	114%
6T6B_MLQ	96%
7P4E_5IV	106%
1FM9_570	119%
1I7I_AZ2	109%
1KNU_YPA	108%
1NYX_DRF	117%
1ZGY_BRL	100%
6ICJ_A0L	93%
4e4k_RRG	33%
5gto_T35	30%
6izm_B0X	36%
8cpi_WY1	42%
2yfe_YFE	39%
3h0a_D30	34%
3ho0_DKD	33%
3hod_ZZH	40%
3vn2_TLS	43%
3B1M_KRC	37%

Table S2. Validation Set of PPARγ Ligands Curated from the ChEMBL Database

Comprehensive collection of 1,561 PPARγ ligands extracted from 4,698 experimental records in the ChEMBL database for human PPARγ activity. The table includes ChEMBL IDs, compound structures, and maximal activation efficacy values (Emax) normalized to rosiglitazone (Emax = 100%). After filtering non-quantitative data and harmonizing efficacy measurements across different reference standards through cross-study comparisons, the final validated dataset comprised 1,079 ligands (393 full agonists and 686 partial agonists) with well-documented pharmacological profiles. This validation set encompasses diverse chemical scaffolds including non-covalent binders, carboxylic acids, and sulfonamides, representing compounds without resolved co-crystal structures and providing a comprehensive chemical and pharmacological scope for evaluating the Ligand Drift Rate descriptor beyond the initial PDB-derived training set.

Molecule ChEMBL ID	Relative to Rosiglitazone
CHEMBL1017	43.16%
CHEMBL107367	119.26%
CHEMBL107498	60.00%
CHEMBL1075723	32.00%
CHEMBL1075724	26.00%
CHEMBL1075732	48.00%
CHEMBL1075894	20.00%
CHEMBL1075895	27.00%
CHEMBL1079669	23.00%
CHEMBL1079961	57.00%
CHEMBL1079962	52.00%
CHEMBL1080140	41.00%
CHEMBL1080365	24.00%
CHEMBL1080699	0.00%
CHEMBL1081003	39.00%
CHEMBL1081786	47.00%
CHEMBL1081950	26.00%
CHEMBL1089120	97.89%
CHEMBL1089121	81.05%
CHEMBL1089859	37.89%
CHEMBL1090521	46.32%
CHEMBL1090549	45.26%
CHEMBL1090550	44.21%
CHEMBL1090870	35.79%
CHEMBL1091530	47.37%
CHEMBL1091572	52.63%
CHEMBL1092387	49.47%
CHEMBL1092559	35.79%
CHEMBL1092633	48.42%
CHEMBL1092634	20.00%
CHEMBL1092854	56.84%
CHEMBL1092855	42.11%
CHEMBL1092876	53.68%
CHEMBL1092900	58.95%
CHEMBL110371	43.51%
CHEMBL113301	15.03%

Molecule ChEMBL ID	Relative to Rosiglitazone
CHEMBL1160899	17.67%
CHEMBL116671	96.00%
CHEMBL1169685	58.00%
CHEMBL1169712	33.00%
CHEMBL1169713	54.00%
CHEMBL1170076	58.88%
CHEMBL1170264	106.36%
CHEMBL1170852	109.21%
CHEMBL1170853	37.00%
CHEMBL1171046	25.00%
CHEMBL1171456	76.00%
CHEMBL1171831	61.73%
CHEMBL1171834	67.43%
CHEMBL1171856	65.00%
CHEMBL1172000	120.61%
CHEMBL1172001	75.02%
CHEMBL1172002	65.53%
CHEMBL1172003	56.03%
CHEMBL1172016	52.23%
CHEMBL1172019	67.43%
CHEMBL1172425	66.00%
CHEMBL1172426	40.00%
CHEMBL1172610	50.00%
CHEMBL1172833	85.00%
CHEMBL1172961	55.00%
CHEMBL1172962	45.00%
CHEMBL1172963	35.00%
CHEMBL1173101	89.00%
CHEMBL1173117	50.00%
CHEMBL1173118	35.00%
CHEMBL1173165	65.00%
CHEMBL1173376	59.83%
CHEMBL1173499	54.13%
CHEMBL1173559	57.00%
CHEMBL1173623	71.00%
CHEMBL1204498	80.00%

Molecule ChEMBL ID	Relative to Rosiglitazone
CHEMBL1209109	0.00%
CHEMBL1209170	10.00%
CHEMBL1209173	0.00%
CHEMBL121	100.00%
CHEMBL1214744	47.01%
CHEMBL1214826	75.02%
CHEMBL1214887	48.43%
CHEMBL1231733	44.34%
CHEMBL1231797	20.89%
CHEMBL1236924	23.94%
CHEMBL144112	162.00%
CHEMBL144489	118.00%
CHEMBL147721	103.00%
CHEMBL148319	18.50%
CHEMBL1641708	18.04%
CHEMBL1641710	35.14%
CHEMBL1641713	1.80%
CHEMBL1641718	1.14%
CHEMBL1644298	31.40%
CHEMBL1644299	85.30%
CHEMBL1644300	58.40%
CHEMBL1644301	64.70%
CHEMBL1644302	58.50%
CHEMBL1644303	43.40%
CHEMBL1644304	52.10%
CHEMBL1644305	89.50%
CHEMBL1644306	67.20%
CHEMBL1644307	72.80%
CHEMBL1644308	44.70%
CHEMBL1644309	18.10%
CHEMBL1644310	61.20%
CHEMBL1644311	45.90%
CHEMBL1644312	61.80%
CHEMBL1644313	53.30%
CHEMBL1644314	32.10%
CHEMBL1644315	29.40%

Molecule ChEMBL ID	Relative to Rosiglitazone
CHEMBL165484	108.70%
CHEMBL165539	78.70%
CHEMBL166408	32.70%
CHEMBL166539	127.00%
CHEMBL166572	106.00%
CHEMBL166628	113.00%
CHEMBL166637	116.00%
CHEMBL166687	85.70%
CHEMBL167043	117.30%
CHEMBL167084	80.00%
CHEMBL167352	125.80%
CHEMBL167378	41.30%
CHEMBL167430	109.70%
CHEMBL167963	126.50%
CHEMBL168033	78.30%
CHEMBL168166	101.00%
CHEMBL1729254	25.00%
CHEMBL178236	36.00%
CHEMBL178245	25.00%
CHEMBL178380	17.39%
CHEMBL1784944	79.00%
CHEMBL1784945	74.00%
CHEMBL1784948	134.00%
CHEMBL1784949	141.00%
CHEMBL1784954	35.00%
CHEMBL179330	26.00%
CHEMBL179542	67.00%
CHEMBL179604	86.00%
CHEMBL1801712	26.09%
CHEMBL1801713	21.74%
CHEMBL1801714	26.09%
CHEMBL1801734	20.65%
CHEMBL1801735	26.09%
CHEMBL1801736	25.00%
CHEMBL1801737	27.17%
CHEMBL1801738	28.26%

Molecule ChEMBL ID	Relative to Rosiglitazone
CHEMBL1801739	25.00%
CHEMBL1801740	33.70%
CHEMBL1801741	27.17%
CHEMBL1801742	30.43%
CHEMBL1801743	29.35%
CHEMBL1801744	35.87%
CHEMBL180430	17.00%
CHEMBL180633	19.00%
CHEMBL180954	0.40%
CHEMBL181087	50.00%
CHEMBL181141	59.00%
CHEMBL181219	33.00%
CHEMBL1813003	107.00%
CHEMBL1813004	99.00%
CHEMBL1813005	88.00%
CHEMBL1813006	120.00%
CHEMBL1813007	104.00%
CHEMBL1813008	104.00%
CHEMBL1813009	87.00%
CHEMBL1813010	192.00%
CHEMBL1813011	165.00%
CHEMBL181554	24.00%
CHEMBL181628	83.00%
CHEMBL1817687	43.68%
CHEMBL181937	97.00%
CHEMBL1819390	67.43%
CHEMBL1819391	90.22%
CHEMBL1819392	69.33%
CHEMBL1819393	75.02%
CHEMBL1819394	94.97%
CHEMBL1819396	92.12%
CHEMBL1819397	44.63%
CHEMBL1819478	81.67%
CHEMBL1819481	65.53%
CHEMBL1819482	57.93%
CHEMBL1819483	121.56%

Molecule ChEMBL ID	Relative to Rosiglitazone
CHEMBL182100	53.50%
CHEMBL182583	44.00%
CHEMBL1828826	25.00%
CHEMBL182941	30.00%
CHEMBL183191	7.00%
CHEMBL183213	5.00%
CHEMBL183502	60.00%
CHEMBL183667	7.00%
CHEMBL183909	16.00%
CHEMBL186179	114.00%
CHEMBL189116	61.20%
CHEMBL190368	18.80%
CHEMBL190905	8.00%
CHEMBL191060	116.00%
CHEMBL191275	57.63%
CHEMBL192518	51.57%
CHEMBL1933093	107.37%
CHEMBL1935491	95.79%
CHEMBL1935492	101.05%
CHEMBL1935493	109.47%
CHEMBL1935494	102.11%
CHEMBL1935495	108.42%
CHEMBL1935496	81.05%
CHEMBL1935497	96.84%
CHEMBL1935498	94.74%
CHEMBL1935499	103.16%
CHEMBL1935500	86.32%
CHEMBL1935501	125.26%
CHEMBL1935607	93.68%
CHEMBL1935608	89.47%
CHEMBL1935609	103.16%
CHEMBL1935610	101.05%
CHEMBL1935611	98.95%
CHEMBL1935612	111.58%
CHEMBL1935613	110.53%
CHEMBL1935614	88.42%

Molecule ChEMBL ID	Relative to Rosiglitazone
CHEMBL1935615	64.21%
CHEMBL1935616	109.47%
CHEMBL1935617	113.68%
CHEMBL1935618	106.32%
CHEMBL1935619	90.53%
CHEMBL1935620	104.21%
CHEMBL1944782	51.00%
CHEMBL1944783	44.00%
CHEMBL1945212	50.00%
CHEMBL1945213	29.00%
CHEMBL1945214	32.00%
CHEMBL1945215	22.00%
CHEMBL1945216	55.00%
CHEMBL1945218	79.00%
CHEMBL1945219	109.00%
CHEMBL1945220	99.00%
CHEMBL1945221	105.00%
CHEMBL1946409	99.73%
CHEMBL1946566	17.00%
CHEMBL1946567	21.00%
CHEMBL1946568	31.30%
CHEMBL1946570	23.42%
CHEMBL1946571	25.00%
CHEMBL1946572	53.00%
CHEMBL1946753	37.00%
CHEMBL1946754	36.00%
CHEMBL1946755	28.00%
CHEMBL1946756	39.00%
CHEMBL1946757	43.00%
CHEMBL1946758	31.00%
CHEMBL1946952	4.00%
CHEMBL1946953	26.00%
CHEMBL194698	16.00%
CHEMBL1947140	45.00%
CHEMBL1947142	28.00%
CHEMBL1947143	27.26%

Molecule ChEMBL ID	Relative to Rosiglitazone
CHEMBL1947144	71.68%
CHEMBL1947145	101.00%
CHEMBL1947146	21.49%
CHEMBL1947147	25.20%
CHEMBL1956349	49.38%
CHEMBL1956350	54.13%
CHEMBL1956357	55.08%
CHEMBL1956358	31.34%
CHEMBL1956359	24.69%
CHEMBL1956360	41.79%
CHEMBL1956361	12.35%
CHEMBL1956362	79.77%
CHEMBL1956363	65.53%
CHEMBL1956364	66.48%
CHEMBL1956365	37.99%
CHEMBL1956366	19.94%
CHEMBL1956367	56.03%
CHEMBL1956368	78.82%
CHEMBL1956369	18.99%
CHEMBL1956370	10.45%
CHEMBL1956371	22.79%
CHEMBL196139	19.00%
CHEMBL196227	13.00%
CHEMBL196260	31.00%
CHEMBL196261	29.00%
CHEMBL1967191	15.00%
CHEMBL197268	42.00%
CHEMBL197684	42.00%
CHEMBL198034	27.00%
CHEMBL198163	100.00%
CHEMBL198166	58.00%
CHEMBL198170	34.00%
CHEMBL198708	26.00%
CHEMBL199121	51.00%
CHEMBL199125	41.00%
CHEMBL199172	25.00%

Molecule ChEMBL ID	Relative to Rosiglitazone
CHEMBL2031856	55.00%
CHEMBL2031859	34.17%
CHEMBL2031860	56.67%
CHEMBL2031954	22.50%
CHEMBL2031956	81.67%
CHEMBL2031957	56.67%
CHEMBL2042802	44.34%
CHEMBL2042803	36.79%
CHEMBL2042804	34.91%
CHEMBL2042805	20.75%
CHEMBL2042806	53.77%
CHEMBL2042807	55.66%
CHEMBL2042808	24.53%
CHEMBL2042809	41.51%
CHEMBL2042810	47.17%
CHEMBL2042811	36.79%
CHEMBL2042812	26.42%
CHEMBL2042813	37.74%
CHEMBL2042814	49.92%
CHEMBL205434	76.00%
CHEMBL207570	30.00%
CHEMBL210162	113.00%
CHEMBL2103824	10.22%
CHEMBL2104753	126.00%
CHEMBL2113104	24.00%
CHEMBL2113105	13.00%
CHEMBL2113106	141.00%
CHEMBL2113107	85.00%
CHEMBL2113108	62.00%
CHEMBL2113109	85.00%
CHEMBL214719	35.50%
CHEMBL217118	20.00%
CHEMBL217907	104.35%
CHEMBL2180631	56.98%
CHEMBL2180634	121.56%
CHEMBL218117	122.83%

Molecule ChEMBL ID	Relative to Rosiglitazone
CHEMBL218950	69.57%
CHEMBL219005	86.96%
CHEMBL219773	56.00%
CHEMBL219824	72.00%
CHEMBL219836	55.00%
CHEMBL2203301	5.70%
CHEMBL2204683	21.84%
CHEMBL2204686	18.04%
CHEMBL2204687	11.40%
CHEMBL2204688	31.34%
CHEMBL2204689	12.35%
CHEMBL2204690	12.35%
CHEMBL2204691	4.75%
CHEMBL2204692	34.19%
CHEMBL2204693	17.09%
CHEMBL2204694	30.39%
CHEMBL2204695	19.94%
CHEMBL2204696	18.89%
CHEMBL2204697	34.19%
CHEMBL2204698	33.24%
CHEMBL2204699	7.60%
CHEMBL2204701	19.94%
CHEMBL2204702	19.94%
CHEMBL220533	111.96%
CHEMBL220597	114.13%
CHEMBL220622	92.00%
CHEMBL2206302	59.00%
CHEMBL2206303	32.00%
CHEMBL2206304	37.00%
CHEMBL2206305	23.00%
CHEMBL2206306	31.00%
CHEMBL220708	101.09%
CHEMBL221123	101.09%
CHEMBL221184	104.35%
CHEMBL221474	96.74%
CHEMBL221641	103.26%

Molecule ChEMBL ID	Relative to Rosiglitazone
CHEMBL221649	125.00%
CHEMBL221850	126.09%
CHEMBL222107	113.04%
CHEMBL222117	105.43%
CHEMBL223128	85.00%
CHEMBL223349	52.00%
CHEMBL223350	70.00%
CHEMBL223351	57.00%
CHEMBL223379	24.00%
CHEMBL223434	76.00%
CHEMBL2260190	6.30%
CHEMBL2260191	3.60%
CHEMBL2260192	2.80%
CHEMBL2260193	6.80%
CHEMBL2260194	7.30%
CHEMBL2260195	5.70%
CHEMBL2260196	1.80%
CHEMBL2260197	4.70%
CHEMBL2260198	8.00%
CHEMBL2260200	5.70%
CHEMBL2312700	21.00%
CHEMBL2312701	28.00%
CHEMBL2312702	33.00%
CHEMBL2312703	12.00%
CHEMBL2312704	8.00%
CHEMBL2312705	11.00%
CHEMBL2312706	7.00%
CHEMBL2312707	34.00%
CHEMBL2312708	47.00%
CHEMBL2312709	18.00%
CHEMBL2312710	13.00%
CHEMBL2312711	16.00%
CHEMBL2312712	47.00%
CHEMBL2312713	43.00%
CHEMBL2312714	42.00%
CHEMBL2312715	49.00%

Molecule ChEMBL ID	Relative to Rosiglitazone
CHEMBL2312716	37.00%
CHEMBL2312717	32.00%
CHEMBL2312718	10.00%
CHEMBL2322172	34.78%
CHEMBL2322174	15.22%
CHEMBL2322175	34.78%
CHEMBL2322176	29.35%
CHEMBL2322437	27.17%
CHEMBL2322439	29.35%
CHEMBL2322441	25.00%
CHEMBL2322442	28.26%
CHEMBL2322443	23.91%
CHEMBL2322444	21.74%
CHEMBL2322445	26.09%
CHEMBL2322446	27.17%
CHEMBL2322447	32.61%
CHEMBL2322448	21.74%
CHEMBL2322449	22.83%
CHEMBL2322450	20.65%
CHEMBL2322451	16.30%
CHEMBL232798	117.00%
CHEMBL232799	138.00%
CHEMBL233003	177.00%
CHEMBL2331559	33.00%
CHEMBL2331770	0.00%
CHEMBL2331771	45.00%
CHEMBL2331772	16.00%
CHEMBL2331773	80.00%
CHEMBL2331774	15.00%
CHEMBL2331775	30.00%
CHEMBL2331777	40.00%
CHEMBL2331778	40.00%
CHEMBL2331779	0.00%
CHEMBL2331780	0.00%
CHEMBL2331781	18.00%
CHEMBL2331782	10.00%

Molecule ChEMBL ID	Relative to Rosiglitazone
CHEMBL2331783	18.00%
CHEMBL2331785	18.00%
CHEMBL2331786	25.00%
CHEMBL2331787	18.00%
CHEMBL233785	182.00%
CHEMBL2338472	30.00%
CHEMBL2338474	21.00%
CHEMBL2338475	42.00%
CHEMBL2338476	18.00%
CHEMBL2338477	10.00%
CHEMBL2338478	55.00%
CHEMBL2338479	20.00%
CHEMBL2338480	25.00%
CHEMBL2338481	20.00%
CHEMBL2338482	25.00%
CHEMBL2338483	10.00%
CHEMBL2338484	13.00%
CHEMBL2338485	10.00%
CHEMBL2338486	15.00%
CHEMBL2338487	0.00%
CHEMBL2338488	0.00%
CHEMBL234819	159.00%
CHEMBL234820	190.00%
CHEMBL234858	118.00%
CHEMBL234859	145.00%
CHEMBL234907	23.00%
CHEMBL234908	28.00%
CHEMBL2377521	95.70%
CHEMBL2377522	72.70%
CHEMBL2377523	95.00%
CHEMBL2377524	99.70%
CHEMBL2377525	104.00%
CHEMBL2377526	76.00%
CHEMBL2377527	113.00%
CHEMBL2377528	100.00%
CHEMBL2377529	108.00%

Molecule ChEMBL ID	Relative to Rosiglitazone
CHEMBL2377530	65.00%
CHEMBL2377531	58.20%
CHEMBL2377532	82.70%
CHEMBL24038	117.10%
CHEMBL2424946	14.00%
CHEMBL2424947	12.00%
CHEMBL2424948	17.00%
CHEMBL2424949	15.00%
CHEMBL2424950	11.00%
CHEMBL2424951	10.00%
CHEMBL2424952	12.00%
CHEMBL2424953	14.00%
CHEMBL2424954	13.00%
CHEMBL2424955	14.00%
CHEMBL2424956	16.00%
CHEMBL24458	109.00%
CHEMBL245656	102.50%
CHEMBL245674	20.00%
CHEMBL246270	93.00%
CHEMBL246272	94.50%
CHEMBL246473	114.50%
CHEMBL247719	32.00%
CHEMBL247720	106.00%
CHEMBL247925	111.00%
CHEMBL247926	68.00%
CHEMBL248530	76.50%
CHEMBL248906	97.50%
CHEMBL25259	123.00%
CHEMBL253952	133.00%
CHEMBL255930	96.00%
CHEMBL264374	64.26%
CHEMBL282686	108.50%
CHEMBL293499	27.16%
CHEMBL294807	6.00%
CHEMBL295416	42.17%
CHEMBL3115236	29.00%

Molecule ChEMBL ID	Relative to Rosiglitazone
CHEMBL3115237	52.00%
CHEMBL3115238	47.00%
CHEMBL3115239	29.00%
CHEMBL3115240	39.00%
CHEMBL3115241	37.00%
CHEMBL3115242	43.00%
CHEMBL3115243	81.00%
CHEMBL3115244	24.00%
CHEMBL3115245	21.00%
CHEMBL3115246	40.00%
CHEMBL3115247	40.00%
CHEMBL314483	75.00%
CHEMBL315048	94.00%
CHEMBL3262946	37.04%
CHEMBL3263231	128.21%
CHEMBL3263232	217.47%
CHEMBL3263233	42.74%
CHEMBL3263236	410.26%
CHEMBL3263237	377.02%
CHEMBL3263244	255.46%
CHEMBL3263245	37.99%
CHEMBL3263246	606.84%
CHEMBL3263247	119.66%
CHEMBL3263248	53.18%
CHEMBL3263249	23.74%
CHEMBL327081	60.13%
CHEMBL327481	72.00%
CHEMBL328099	101.00%
CHEMBL328455	101.00%
CHEMBL3318036	135.80%
CHEMBL3318037	128.21%
CHEMBL3318038	119.66%
CHEMBL3318039	111.11%
CHEMBL3318040	138.65%
CHEMBL3318041	150.05%
CHEMBL3335622	330.48%

Molecule ChEMBL ID	Relative to Rosiglitazone
CHEMBL3335623	364.67%
CHEMBL3335624	55.08%
CHEMBL3335625	209.88%
CHEMBL3335628	227.92%
CHEMBL3335629	108.26%
CHEMBL3335653	114.91%
CHEMBL3335654	166.19%
CHEMBL3335655	154.80%
CHEMBL3335656	66.48%
CHEMBL3335657	146.25%
CHEMBL3335658	698.01%
CHEMBL3335659	146.25%
CHEMBL3335660	93.07%
CHEMBL3335661	91.17%
CHEMBL3335662	93.07%
CHEMBL3335663	77.87%
CHEMBL3335666	125.36%
CHEMBL3335667	243.11%
CHEMBL3335672	76.92%
CHEMBL3348830	0.00%
CHEMBL3352822	52.00%
CHEMBL3355859	75.00%
CHEMBL3355860	65.00%
CHEMBL3355861	50.00%
CHEMBL3355862	56.00%
CHEMBL3355863	77.00%
CHEMBL3355864	69.00%
CHEMBL3355865	46.00%
CHEMBL3355866	54.00%
CHEMBL3355867	31.00%
CHEMBL3355868	59.00%
CHEMBL3355869	54.00%
CHEMBL3355870	24.00%
CHEMBL3355871	61.00%
CHEMBL3355872	45.00%
CHEMBL3358036	35.08%

Molecule ChEMBL ID	Relative to Rosiglitazone
CHEMBL3393177	394.11%
CHEMBL3393178	398.86%
CHEMBL3393179	152.90%
CHEMBL3393180	66.48%
CHEMBL3393181	118.71%
CHEMBL3393182	189.93%
CHEMBL3393183	150.05%
CHEMBL3393185	12.54%
CHEMBL3393187	318.14%
CHEMBL339359	113.96%
CHEMBL3397548	38.00%
CHEMBL3397549	44.00%
CHEMBL3397550	30.00%
CHEMBL3397551	51.00%
CHEMBL3397552	54.00%
CHEMBL3397553	50.00%
CHEMBL3397554	19.00%
CHEMBL3397555	41.00%
CHEMBL3397556	36.00%
CHEMBL3397557	44.00%
CHEMBL3397558	26.00%
CHEMBL3397559	26.00%
CHEMBL3397560	20.00%
CHEMBL3397561	27.00%
CHEMBL3397562	11.00%
CHEMBL3397563	19.00%
CHEMBL3397709	72.17%
CHEMBL3397710	102.56%
CHEMBL3397711	53.18%
CHEMBL3397715	72.17%
CHEMBL3397716	53.18%
CHEMBL3397717	97.82%
CHEMBL3397718	113.96%
CHEMBL3397724	62.68%
CHEMBL3397726	92.12%
CHEMBL3397727	150.05%

Molecule ChEMBL ID	Relative to Rosiglitazone
CHEMBL3398423	83.00%
CHEMBL3398424	58.00%
CHEMBL3398425	35.00%
CHEMBL3398427	33.00%
CHEMBL3398429	99.00%
CHEMBL3398430	85.00%
CHEMBL3398431	7.00%
CHEMBL3398432	104.00%
CHEMBL3398433	51.00%
CHEMBL3398435	33.00%
CHEMBL3398436	66.50%
CHEMBL3398438	35.50%
CHEMBL3398439	9.00%
CHEMBL3398440	105.00%
CHEMBL3398441	59.00%
CHEMBL3398442	76.00%
CHEMBL3398443	99.00%
CHEMBL3398444	26.50%
CHEMBL3398446	75.00%
CHEMBL3398448	67.00%
CHEMBL3398449	49.00%
CHEMBL3398453	52.00%
CHEMBL3398455	26.00%
CHEMBL3398456	126.00%
CHEMBL3398457	45.00%
CHEMBL3398458	64.00%
CHEMBL3398459	62.00%
CHEMBL3425533	11.30%
CHEMBL345009	102.00%
CHEMBL3545278	10.45%
CHEMBL3545280	100.00%
CHEMBL354863	80.30%
CHEMBL355695	118.30%
CHEMBL3585572	100.00%
CHEMBL3585573	33.00%
CHEMBL3585574	30.00%

Molecule ChEMBL ID	Relative to Rosiglitazone
CHEMBL3585575	70.00%
CHEMBL3585576	30.00%
CHEMBL3585577	22.50%
CHEMBL3589163	48.74%
CHEMBL359476	42.00%
CHEMBL359548	34.00%
CHEMBL3597313	31.30%
CHEMBL3597314	16.80%
CHEMBL3597386	36.50%
CHEMBL3597387	23.60%
CHEMBL3597388	113.50%
CHEMBL3597389	79.60%
CHEMBL3597390	102.80%
CHEMBL3597391	93.40%
CHEMBL3597392	100.80%
CHEMBL3597393	81.20%
CHEMBL3597394	85.70%
CHEMBL3597395	90.90%
CHEMBL3597396	73.40%
CHEMBL3597397	16.10%
CHEMBL3597398	75.10%
CHEMBL3597399	89.20%
CHEMBL3597400	134.40%
CHEMBL3597401	104.50%
CHEMBL3597402	109.40%
CHEMBL3597403	100.50%
CHEMBL3597404	91.70%
CHEMBL3597405	99.40%
CHEMBL3597406	92.60%
CHEMBL3597407	83.70%
CHEMBL3597408	19.20%
CHEMBL3597409	84.30%
CHEMBL3597410	81.80%
CHEMBL3597411	130.30%
CHEMBL3597412	122.50%
CHEMBL3597413	92.50%

Molecule ChEMBL ID	Relative to Rosiglitazone
CHEMBL3597414	145.40%
CHEMBL3597415	106.40%
CHEMBL3597416	119.30%
CHEMBL3597417	89.70%
CHEMBL3597418	71.20%
CHEMBL3597419	118.80%
CHEMBL3597420	99.80%
CHEMBL3597421	85.20%
CHEMBL3597422	71.10%
CHEMBL3597423	73.10%
CHEMBL3597424	101.60%
CHEMBL3597425	66.90%
CHEMBL3597426	100.30%
CHEMBL3597427	94.60%
CHEMBL3597428	101.90%
CHEMBL3597429	77.70%
CHEMBL3597430	77.90%
CHEMBL3597431	92.70%
CHEMBL3597432	109.60%
CHEMBL3597433	110.30%
CHEMBL3597434	104.40%
CHEMBL3597435	120.10%
CHEMBL360420	10.60%
CHEMBL361351	7.00%
CHEMBL361395	75.00%
CHEMBL361617	14.00%
CHEMBL361773	27.00%
CHEMBL363805	31.00%
CHEMBL364254	4.50%
CHEMBL364748	71.31%
CHEMBL366157	7.80%
CHEMBL367360	97.00%
CHEMBL3695775	0.00%
CHEMBL3695811	60.00%
CHEMBL3695816	36.00%
CHEMBL369890	11.50%

Molecule ChEMBL ID	Relative to Rosiglitazone
CHEMBL370054	9.70%
CHEMBL370152	25.00%
CHEMBL370374	32.00%
CHEMBL3707395	105.00%
CHEMBL371120	76.50%
CHEMBL371320	47.10%
CHEMBL373318	45.00%
CHEMBL373685	80.00%
CHEMBL373874	106.52%
CHEMBL374296	75.00%
CHEMBL374323	94.57%
CHEMBL374344	84.00%
CHEMBL374448	141.30%
CHEMBL3751930	42.00%
CHEMBL3751969	89.00%
CHEMBL3752362	22.00%
CHEMBL3752643	25.00%
CHEMBL3752940	78.00%
CHEMBL3753029	65.00%
CHEMBL3753640	30.00%
CHEMBL3753786	34.00%
CHEMBL3753819	44.00%
CHEMBL3753947	38.00%
CHEMBL3753965	55.00%
CHEMBL3754569	18.00%
CHEMBL3754669	54.00%
CHEMBL376325	107.61%
CHEMBL3763278	50.33%
CHEMBL3763373	100.66%
CHEMBL3763432	72.17%
CHEMBL3763491	116.81%
CHEMBL3763887	112.06%
CHEMBL3764004	83.57%
CHEMBL3764076	171.89%
CHEMBL3764102	182.34%
CHEMBL3764209	104.46%

Molecule ChEMBL ID	Relative to Rosiglitazone
CHEMBL3764216	150.05%
CHEMBL3764398	151.95%
CHEMBL3764424	104.46%
CHEMBL3764450	125.36%
CHEMBL3764453	64.58%
CHEMBL3764546	64.58%
CHEMBL376460	61.00%
CHEMBL3764607	56.98%
CHEMBL3764773	81.67%
CHEMBL3764792	129.15%
CHEMBL3764795	85.47%
CHEMBL3764848	111.11%
CHEMBL3764891	162.39%
CHEMBL3764948	170.94%
CHEMBL3765141	120.61%
CHEMBL3765347	89.27%
CHEMBL3765360	70.28%
CHEMBL3765679	133.90%
CHEMBL3765690	104.46%
CHEMBL3765763	118.71%
CHEMBL379439	91.00%
CHEMBL3818976	64.58%
CHEMBL383607	72.00%
CHEMBL38508	179.00%
CHEMBL386957	96.74%
CHEMBL387133	78.26%
CHEMBL387373	56.00%
CHEMBL38943	61.10%
CHEMBL3904906	52.61%
CHEMBL3905419	31.15%
CHEMBL3907308	20.61%
CHEMBL3912543	54.99%
CHEMBL3914401	31.43%
CHEMBL3916286	37.99%
CHEMBL393005	160.00%
CHEMBL393381	197.00%

Molecule ChEMBL ID	Relative to Rosiglitazone
CHEMBL3934455	28.77%
CHEMBL394031	113.00%
CHEMBL3947936	42.45%
CHEMBL394947	73.50%
CHEMBL394948	61.00%
CHEMBL394967	71.50%
CHEMBL394968	97.50%
CHEMBL395191	97.50%
CHEMBL3958255	71.23%
CHEMBL396109	119.00%
CHEMBL3962742	35.90%
CHEMBL396694	58.00%
CHEMBL396696	68.50%
CHEMBL397120	105.50%
CHEMBL3976173	78.35%
CHEMBL3977198	79.87%
CHEMBL3984220	54.51%
CHEMBL3985281	15.38%
CHEMBL3986047	10.45%
CHEMBL398804	187.00%
CHEMBL401015	204.00%
CHEMBL4059763	8.26%
CHEMBL4060007	38.00%
CHEMBL4062472	36.00%
CHEMBL4062724	36.00%
CHEMBL4063529	34.00%
CHEMBL4064900	26.00%
CHEMBL4066332	28.49%
CHEMBL4066489	65.43%
CHEMBL4066889	70.00%
CHEMBL4067479	49.00%
CHEMBL4068424	29.44%
CHEMBL4069460	30.58%
CHEMBL4069463	123.46%
CHEMBL4071373	39.00%
CHEMBL4072125	39.00%

Molecule ChEMBL ID	Relative to Rosiglitazone
CHEMBL4072143	36.00%
CHEMBL4072395	25.00%
CHEMBL4073181	46.00%
CHEMBL4073499	26.00%
CHEMBL4074391	43.00%
CHEMBL4074565	3.00%
CHEMBL4075090	9.31%
CHEMBL4075210	36.09%
CHEMBL4075456	42.00%
CHEMBL4078107	90.88%
CHEMBL4078402	58.50%
CHEMBL4079308	50.00%
CHEMBL408	83.23%
CHEMBL4080865	70.37%
CHEMBL4081907	46.00%
CHEMBL4083589	48.00%
CHEMBL4084839	9.00%
CHEMBL4086261	68.57%
CHEMBL4087052	39.00%
CHEMBL4088048	11.40%
CHEMBL4088168	67.00%
CHEMBL4089775	35.00%
CHEMBL4090484	58.21%
CHEMBL4090489	41.79%
CHEMBL4090691	45.00%
CHEMBL4091374	96.91%
CHEMBL4091398	10.45%
CHEMBL4092600	17.00%
CHEMBL4093146	48.00%
CHEMBL4093864	30.00%
CHEMBL4094026	65.62%
CHEMBL4094877	46.00%
CHEMBL4095031	10.00%
CHEMBL4095614	104.46%
CHEMBL4095771	87.37%
CHEMBL4095821	38.00%

Molecule ChEMBL ID	Relative to Rosiglitazone
CHEMBL4095891	52.00%
CHEMBL4096160	103.89%
CHEMBL4096447	104.56%
CHEMBL4098202	37.99%
CHEMBL4098415	32.00%
CHEMBL4100336	20.00%
CHEMBL4101596	45.00%
CHEMBL4101759	34.95%
CHEMBL4102794	10.00%
CHEMBL4103575	32.00%
CHEMBL4104178	66.19%
CHEMBL4104907	74.00%
CHEMBL4105094	45.39%
CHEMBL414836	30.43%
CHEMBL4159072	71.00%
CHEMBL4159590	67.00%
CHEMBL4159720	74.00%
CHEMBL4159730	79.00%
CHEMBL4159798	125.00%
CHEMBL4159849	83.00%
CHEMBL4159869	114.00%
CHEMBL4160416	98.00%
CHEMBL4160620	78.00%
CHEMBL4160661	38.00%
CHEMBL4160935	54.00%
CHEMBL4160945	126.00%
CHEMBL4161305	80.00%
CHEMBL4161332	88.00%
CHEMBL4161352	86.00%
CHEMBL4161407	62.00%
CHEMBL4161693	52.00%
CHEMBL4161810	78.00%
CHEMBL4161905	80.00%
CHEMBL4162387	75.00%
CHEMBL4162757	86.00%
CHEMBL4162863	83.00%

Molecule ChEMBL ID	Relative to Rosiglitazone
CHEMBL4162908	79.00%
CHEMBL4162949	40.00%
CHEMBL4163066	95.00%
CHEMBL4163093	79.00%
CHEMBL4163237	103.00%
CHEMBL4163242	107.00%
CHEMBL4164018	84.00%
CHEMBL4164063	70.00%
CHEMBL4164343	112.00%
CHEMBL4164423	68.00%
CHEMBL4164442	111.00%
CHEMBL4164753	82.00%
CHEMBL4164769	79.00%
CHEMBL4164824	75.00%
CHEMBL4164828	117.00%
CHEMBL4165766	42.00%
CHEMBL4165832	58.00%
CHEMBL4165852	56.00%
CHEMBL4166201	73.00%
CHEMBL4166323	61.00%
CHEMBL4166727	65.00%
CHEMBL4166946	55.00%
CHEMBL4167180	73.00%
CHEMBL4167211	73.00%
CHEMBL4167504	40.00%
CHEMBL4167706	93.00%
CHEMBL4167709	90.00%
CHEMBL4167731	92.00%
CHEMBL4167767	74.00%
CHEMBL4167789	223.00%
CHEMBL4167873	84.00%
CHEMBL4168205	71.00%
CHEMBL4168263	80.00%
CHEMBL4168900	93.00%
CHEMBL4169260	61.00%
CHEMBL4169279	81.00%

Molecule ChEMBL ID	Relative to Rosiglitazone
CHEMBL4169333	60.00%
CHEMBL4169557	92.00%
CHEMBL4169731	92.00%
CHEMBL4170144	81.00%
CHEMBL4170351	67.00%
CHEMBL4170594	64.00%
CHEMBL4170847	60.00%
CHEMBL4170881	118.00%
CHEMBL4171153	139.00%
CHEMBL4171415	70.00%
CHEMBL4171548	66.00%
CHEMBL4172034	87.00%
CHEMBL4172322	80.00%
CHEMBL4172668	77.00%
CHEMBL4172725	75.00%
CHEMBL4173064	80.00%
CHEMBL4173286	79.00%
CHEMBL4173802	89.00%
CHEMBL4174598	105.00%
CHEMBL4174671	61.00%
CHEMBL4174713	46.00%
CHEMBL4174837	59.00%
CHEMBL4174996	120.00%
CHEMBL4175074	96.00%
CHEMBL4175461	74.00%
CHEMBL4175728	90.00%
CHEMBL4175921	79.00%
CHEMBL4176058	76.00%
CHEMBL4176105	76.00%
CHEMBL4176443	62.00%
CHEMBL4176459	69.00%
CHEMBL4176512	73.00%
CHEMBL4176762	66.00%
CHEMBL4176819	91.00%
CHEMBL4176938	41.00%
CHEMBL4176941	100.00%

Molecule ChEMBL ID	Relative to Rosiglitazone
CHEMBL4177154	80.00%
CHEMBL420973	104.00%
CHEMBL421729	129.00%
CHEMBL4237252	57.00%
CHEMBL4242324	27.00%
CHEMBL4243327	8.00%
CHEMBL4247103	37.00%
CHEMBL4248276	54.00%
CHEMBL4251393	61.00%
CHEMBL425605	88.00%
CHEMBL425639	111.96%
CHEMBL426550	101.09%
CHEMBL426632	42.40%
CHEMBL426878	103.26%
CHEMBL4277723	8.64%
CHEMBL4277928	12.35%
CHEMBL4278109	9.02%
CHEMBL4279086	34.19%
CHEMBL4279177	56.03%
CHEMBL428019	152.00%
CHEMBL4280282	69.33%
CHEMBL4281564	9.21%
CHEMBL4282451	6.65%
CHEMBL4282648	0.38%
CHEMBL4285849	6.84%
CHEMBL4286027	13.30%
CHEMBL4286347	27.54%
CHEMBL428748	80.50%
CHEMBL4288363	4.56%
CHEMBL4288463	22.79%
CHEMBL4288574	44.63%
CHEMBL4289449	18.04%
CHEMBL4289768	19.94%
CHEMBL4289861	21.84%
CHEMBL4290919	17.09%
CHEMBL4293061	10.45%

Molecule ChEMBL ID	Relative to Rosiglitazone
CHEMBL4293242	1.42%
CHEMBL4294239	17.09%
CHEMBL429823	103.50%
CHEMBL431348	19.94%
CHEMBL435517	24.00%
CHEMBL441554	65.00%
CHEMBL442909	74.00%
CHEMBL4434731	15.00%
CHEMBL4435043	41.98%
CHEMBL4435259	84.52%
CHEMBL4436393	70.28%
CHEMBL4436626	24.00%
CHEMBL4438226	9.00%
CHEMBL443910	173.00%
CHEMBL4439373	31.00%
CHEMBL4439891	14.00%
CHEMBL4440354	68.38%
CHEMBL4443192	37.04%
CHEMBL4443400	23.00%
CHEMBL4443588	108.26%
CHEMBL4443931	56.00%
CHEMBL4444322	10.00%
CHEMBL4444760	62.68%
CHEMBL4444888	95.92%
CHEMBL4445012	24.69%
CHEMBL4445084	95.92%
CHEMBL4445737	8.00%
CHEMBL4446031	69.33%
CHEMBL4446679	18.04%
CHEMBL4447835	75.97%
CHEMBL4448988	76.92%
CHEMBL4449283	17.00%
CHEMBL4449414	6.79%
CHEMBL4449760	24.00%
CHEMBL4452865	58.88%
CHEMBL4453360	82.62%

Molecule ChEMBL ID	Relative to Rosiglitazone
CHEMBL4453610	12.00%
CHEMBL4456647	93.07%
CHEMBL4458208	11.00%
CHEMBL445921	42.00%
CHEMBL4459701	23.00%
CHEMBL4460072	86.00%
CHEMBL4460166	26.59%
CHEMBL4461490	12.00%
CHEMBL4461949	10.00%
CHEMBL4463464	31.62%
CHEMBL4463568	48.00%
CHEMBL4463602	94.02%
CHEMBL446365	66.00%
CHEMBL4463957	87.37%
CHEMBL4464538	32.29%
CHEMBL4464717	99.72%
CHEMBL4465311	116.81%
CHEMBL4465521	30.00%
CHEMBL4466036	51.57%
CHEMBL4466241	85.47%
CHEMBL4466393	60.00%
CHEMBL4466552	37.00%
CHEMBL4466709	63.63%
CHEMBL4468084	64.58%
CHEMBL4468637	77.87%
CHEMBL446893	97.00%
CHEMBL4469716	44.00%
CHEMBL4469751	95.92%
CHEMBL4470753	118.00%
CHEMBL4471255	18.00%
CHEMBL4471421	71.23%
CHEMBL4471853	12.65%
CHEMBL4472191	121.56%
CHEMBL4474407	37.04%
CHEMBL4474657	18.52%
CHEMBL4483378	44.63%

Molecule ChEMBL ID	Relative to Rosiglitazone
CHEMBL449099	74.00%
CHEMBL451321	12.35%
CHEMBL4513559	59.00%
CHEMBL4514313	20.00%
CHEMBL4517263	84.52%
CHEMBL4517866	23.84%
CHEMBL4519376	40.84%
CHEMBL4519542	88.00%
CHEMBL452051	27.00%
CHEMBL452054	36.00%
CHEMBL4520827	0.95%
CHEMBL4521388	108.26%
CHEMBL452299	23.00%
CHEMBL4527674	20.04%
CHEMBL4527893	78.82%
CHEMBL4528726	52.70%
CHEMBL4531918	91.17%
CHEMBL4533202	11.00%
CHEMBL4533822	8.00%
CHEMBL4535401	61.73%
CHEMBL4535730	55.00%
CHEMBL4536821	26.00%
CHEMBL4537335	126.31%
CHEMBL4538895	28.02%
CHEMBL4541281	50.33%
CHEMBL4541559	56.98%
CHEMBL4542891	83.57%
CHEMBL4546069	93.07%
CHEMBL4546437	113.96%
CHEMBL4546798	33.24%
CHEMBL454691	27.00%
CHEMBL4548480	47.48%
CHEMBL4550307	47.40%
CHEMBL4550608	88.32%
CHEMBL455126	32.00%
CHEMBL455127	43.00%

Molecule ChEMBL ID	Relative to Rosiglitazone
CHEMBL455130	19.00%
CHEMBL4551334	19.75%
CHEMBL4552317	41.79%
CHEMBL4555317	42.00%
CHEMBL4555407	12.63%
CHEMBL4556396	96.87%
CHEMBL4557173	12.00%
CHEMBL4558284	16.00%
CHEMBL4558373	46.00%
CHEMBL455853	164.00%
CHEMBL4560767	6.74%
CHEMBL4566395	17.00%
CHEMBL4567211	69.33%
CHEMBL4568299	15.00%
CHEMBL456912	89.00%
CHEMBL457	79.00%
CHEMBL4571498	28.49%
CHEMBL4574542	51.28%
CHEMBL4575077	47.10%
CHEMBL4575123	14.00%
CHEMBL4576586	18.61%
CHEMBL4580572	14.00%
CHEMBL458221	239.00%
CHEMBL4586093	27.00%
CHEMBL458648	122.00%
CHEMBL4586557	44.63%
CHEMBL4587577	14.91%
CHEMBL4587597	42.64%
CHEMBL458866	101.00%
CHEMBL458867	109.00%
CHEMBL4588919	35.00%
CHEMBL4589453	110.16%
CHEMBL4591010	13.68%
CHEMBL4591405	97.82%
CHEMBL4591564	32.00%
CHEMBL4592451	10.00%

Molecule ChEMBL ID	Relative to Rosiglitazone
CHEMBL4592882	11.00%
CHEMBL4592935	1.90%
CHEMBL4593237	19.47%
CHEMBL4593987	95.00%
CHEMBL459466	25.00%
CHEMBL459521	16.00%
CHEMBL459972	70.98%
CHEMBL460072	29.00%
CHEMBL460073	23.00%
CHEMBL461378	37.00%
CHEMBL461379	22.00%
CHEMBL461571	50.85%
CHEMBL461883	57.93%
CHEMBL461944	20.00%
CHEMBL462378	14.00%
CHEMBL469937	48.00%
CHEMBL470144	31.00%
CHEMBL470145	42.00%
CHEMBL471011	60.00%
CHEMBL471191	77.00%
CHEMBL471851	26.00%
CHEMBL472244	22.00%
CHEMBL472245	32.00%
CHEMBL472246	40.00%
CHEMBL472451	46.00%
CHEMBL473952	76.00%
CHEMBL473953	39.00%
CHEMBL473961	72.00%
CHEMBL4740243	99.00%
CHEMBL4740359	76.00%
CHEMBL474148	19.00%
CHEMBL474149	65.00%
CHEMBL474157	82.00%
CHEMBL474158	84.00%
CHEMBL4742137	11.40%
CHEMBL4743099	37.04%

Molecule ChEMBL ID	Relative to Rosiglitazone
CHEMBL4745121	131.00%
CHEMBL4745294	23.74%
CHEMBL474548	49.00%
CHEMBL474550	21.00%
CHEMBL474551	39.00%
CHEMBL4745787	104.00%
CHEMBL4746369	92.00%
CHEMBL4749151	116.00%
CHEMBL4749400	103.00%
CHEMBL474947	87.00%
CHEMBL4749785	44.00%
CHEMBL4751747	31.34%
CHEMBL4751849	86.00%
CHEMBL4752692	36.00%
CHEMBL4752897	71.00%
CHEMBL4753247	83.00%
CHEMBL4755265	18.99%
CHEMBL4756972	36.09%
CHEMBL4757935	110.00%
CHEMBL4758029	110.00%
CHEMBL4759515	95.00%
CHEMBL475988	88.00%
CHEMBL475989	72.00%
CHEMBL4759931	100.00%
CHEMBL4761285	112.00%
CHEMBL476197	75.00%
CHEMBL4762110	45.00%
CHEMBL4762401	19.94%
CHEMBL4762983	103.00%
CHEMBL4763760	94.00%
CHEMBL4764095	99.00%
CHEMBL4764106	74.00%
CHEMBL477095	102.17%
CHEMBL477119	50.20%
CHEMBL477312	50.20%
CHEMBL4776398	77.00%

Molecule ChEMBL ID	Relative to Rosiglitazone
CHEMBL4776412	125.00%
CHEMBL477644	78.00%
CHEMBL4780380	59.00%
CHEMBL4780590	88.00%
CHEMBL4783088	83.00%
CHEMBL4783659	106.00%
CHEMBL4784296	101.00%
CHEMBL4784654	33.24%
CHEMBL4787617	83.00%
CHEMBL4787702	28.49%
CHEMBL478897	62.00%
CHEMBL4789439	99.00%
CHEMBL4789687	66.00%
CHEMBL479207	116.05%
CHEMBL4792723	25.83%
CHEMBL4792982	49.38%
CHEMBL4798903	84.00%
CHEMBL4799629	46.53%
CHEMBL480298	7.00%
CHEMBL482659	38.00%
CHEMBL482823	129.00%
CHEMBL482824	155.00%
CHEMBL482827	161.00%
CHEMBL482857	37.00%
CHEMBL483032	158.00%
CHEMBL483035	95.00%
CHEMBL484063	173.00%
CHEMBL484263	114.00%
CHEMBL484264	102.00%
CHEMBL48435	132.95%
CHEMBL484462	151.00%
CHEMBL4846419	72.17%
CHEMBL4846504	64.96%
CHEMBL4847196	39.03%
CHEMBL4847662	15.18%
CHEMBL4848717	11.02%

Molecule ChEMBL ID	Relative to Rosiglitazone
CHEMBL4848974	59.93%
CHEMBL4850352	57.00%
CHEMBL4850966	0.01%
CHEMBL4852222	2.69%
CHEMBL4852368	20.51%
CHEMBL4852747	30.00%
CHEMBL4852865	3.64%
CHEMBL485291	158.00%
CHEMBL4853605	70.00%
CHEMBL4854359	59.00%
CHEMBL4854804	104.30%
CHEMBL4855306	12.75%
CHEMBL4856081	61.00%
CHEMBL4857550	30.56%
CHEMBL4858086	18.28%
CHEMBL4858856	30.00%
CHEMBL4858879	21.08%
CHEMBL4859407	2.51%
CHEMBL4859651	32.52%
CHEMBL4859884	35.00%
CHEMBL4860127	14.25%
CHEMBL4860595	43.59%
CHEMBL4861711	64.00%
CHEMBL486205	18.00%
CHEMBL4863289	0.10%
CHEMBL4863378	13.96%
CHEMBL486350	34.00%
CHEMBL486357	64.00%
CHEMBL486363	47.00%
CHEMBL486364	61.00%
CHEMBL486365	28.00%
CHEMBL4863779	68.00%
CHEMBL4865460	6.32%
CHEMBL4865961	25.00%
CHEMBL4866406	5.39%
CHEMBL4866568	18.20%

Molecule ChEMBL ID	Relative to Rosiglitazone
CHEMBL4866667	13.11%
CHEMBL4867586	30.58%
CHEMBL486764	20.00%
CHEMBL4868676	15.87%
CHEMBL486892	64.00%
CHEMBL4869429	19.66%
CHEMBL4869870	11.52%
CHEMBL4870332	35.99%
CHEMBL4872298	0.52%
CHEMBL4874383	40.84%
CHEMBL4876536	29.00%
CHEMBL4877893	0.19%
CHEMBL4878808	55.00%
CHEMBL488991	222.00%
CHEMBL489074	40.00%
CHEMBL489075	33.00%
CHEMBL489506	23.00%
CHEMBL489507	20.00%
CHEMBL489559	119.00%
CHEMBL489651	25.00%
CHEMBL489765	141.00%
CHEMBL489766	164.00%
CHEMBL490029	25.00%
CHEMBL490220	163.00%
CHEMBL490425	160.00%
CHEMBL490426	135.00%
CHEMBL490832	214.00%
CHEMBL491142	86.00%
CHEMBL491627	141.00%
CHEMBL491750	85.00%
CHEMBL491751	138.00%
CHEMBL491820	39.00%
CHEMBL491950	25.00%
CHEMBL492046	71.00%
CHEMBL492047	106.00%
CHEMBL495990	43.00%

Molecule ChEMBL ID	Relative to Rosiglitazone
CHEMBL496593	73.00%
CHEMBL496807	20.00%
CHEMBL497203	96.00%
CHEMBL497204	92.00%
CHEMBL499038	26.00%
CHEMBL500257	69.17%
CHEMBL501335	136.00%
CHEMBL501439	23.00%
CHEMBL501440	34.00%
CHEMBL501617	35.00%
CHEMBL501982	65.00%
CHEMBL502521	99.00%
CHEMBL503072	21.00%
CHEMBL506419	81.00%
CHEMBL5070178	115.00%
CHEMBL5071398	21.65%
CHEMBL5071964	35.14%
CHEMBL5071965	83.00%
CHEMBL5072619	149.00%
CHEMBL5073521	84.00%
CHEMBL5074074	218.42%
CHEMBL5074222	34.19%
CHEMBL5074383	71.56%
CHEMBL507477	47.00%
CHEMBL5074984	119.66%
CHEMBL5075443	15.19%
CHEMBL5075652	15.07%
CHEMBL5075710	32.29%
CHEMBL5075917	134.00%
CHEMBL5075951	76.00%
CHEMBL5076063	110.00%
CHEMBL5076090	87.00%
CHEMBL5076679	127.26%
CHEMBL5076771	80.00%
CHEMBL5077172	18.99%
CHEMBL5077363	53.18%

Molecule ChEMBL ID	Relative to Rosiglitazone
CHEMBL5077367	92.00%
CHEMBL5077842	90.00%
CHEMBL5078096	30.39%
CHEMBL5078180	191.83%
CHEMBL5078556	126.00%
CHEMBL5078732	10.45%
CHEMBL5078989	73.12%
CHEMBL5079563	52.23%
CHEMBL5079792	13.30%
CHEMBL5079871	99.00%
CHEMBL5079941	20.89%
CHEMBL5080474	27.54%
CHEMBL5080632	12.35%
CHEMBL5080856	11.40%
CHEMBL5080884	114.00%
CHEMBL5081013	170.94%
CHEMBL5081430	96.00%
CHEMBL5081457	78.82%
CHEMBL5081786	14.25%
CHEMBL5081988	151.00%
CHEMBL5081996	150.00%
CHEMBL5082042	11.40%
CHEMBL508225	76.00%
CHEMBL5083326	29.44%
CHEMBL5083878	15.19%
CHEMBL5084019	87.00%
CHEMBL5084314	132.00%
CHEMBL5084336	105.64%
CHEMBL5084377	80.00%
CHEMBL5084422	27.00%
CHEMBL5084529	17.76%
CHEMBL5084763	98.00%
CHEMBL5085291	8.50%
CHEMBL5085496	180.00%
CHEMBL5085545	22.40%
CHEMBL5085618	31.34%

Molecule ChEMBL ID	Relative to Rosiglitazone
CHEMBL5085675	51.28%
CHEMBL5085903	46.00%
CHEMBL5086086	15.19%
CHEMBL5086358	70.00%
CHEMBL5086718	18.04%
CHEMBL5087046	19.94%
CHEMBL5087115	67.00%
CHEMBL5087157	80.00%
CHEMBL5087313	10.45%
CHEMBL508733	66.00%
CHEMBL5087457	26.59%
CHEMBL5087514	32.00%
CHEMBL5087538	22.79%
CHEMBL5087638	133.00%
CHEMBL5087833	22.79%
CHEMBL5087861	18.04%
CHEMBL5088299	16.14%
CHEMBL5089010	125.00%
CHEMBL5089520	104.00%
CHEMBL5089555	13.00%
CHEMBL5089733	209.00%
CHEMBL5089975	25.45%
CHEMBL5090087	78.82%
CHEMBL5090142	88.00%
CHEMBL5090334	39.89%
CHEMBL5090355	31.34%
CHEMBL5090373	21.84%
CHEMBL5090762	64.58%
CHEMBL5090820	41.79%
CHEMBL5090821	32.29%
CHEMBL5090873	24.69%
CHEMBL5091066	61.73%
CHEMBL5091134	113.01%
CHEMBL5091419	13.00%
CHEMBL5092049	20.89%
CHEMBL509236	27.00%

Molecule ChEMBL ID	Relative to Rosiglitazone
CHEMBL5092961	114.00%
CHEMBL5092964	56.03%
CHEMBL509369	19.00%
CHEMBL5093865	16.14%
CHEMBL5093897	20.89%
CHEMBL5094028	26.59%
CHEMBL5094105	95.00%
CHEMBL5094953	103.51%
CHEMBL510180	35.00%
CHEMBL511100	38.00%
CHEMBL512563	28.00%
CHEMBL513244	25.00%
CHEMBL514007	90.00%
CHEMBL514173	19.00%
CHEMBL514174	21.00%
CHEMBL514328	72.00%
CHEMBL514381	65.00%
CHEMBL514441	75.00%
CHEMBL514568	84.00%
CHEMBL514713	89.00%
CHEMBL515106	70.00%
CHEMBL515454	76.00%
CHEMBL515666	101.00%
CHEMBL515991	263.00%
CHEMBL5171949	21.00%
CHEMBL5172173	30.00%
CHEMBL5173082	30.00%
CHEMBL5173134	12.00%
CHEMBL517436	26.00%
CHEMBL518038	132.00%
CHEMBL5182467	30.00%
CHEMBL5184438	16.00%
CHEMBL518499	21.00%
CHEMBL518507	105.41%
CHEMBL5187974	30.00%
CHEMBL519060	24.00%

Molecule ChEMBL ID	Relative to Rosiglitazone
CHEMBL5191536	17.00%
CHEMBL5191690	17.00%
CHEMBL5194126	14.00%
CHEMBL5194570	20.00%
CHEMBL5195064	17.00%
CHEMBL5196394	22.00%
CHEMBL5196830	22.00%
CHEMBL5198233	30.00%
CHEMBL519857	121.00%
CHEMBL5199782	15.00%
CHEMBL5202264	38.00%
CHEMBL520305	83.00%
CHEMBL5203327	21.00%
CHEMBL5204992	10.00%
CHEMBL5207071	14.00%
CHEMBL5207420	33.00%
CHEMBL521289	155.00%
CHEMBL521796	135.00%
CHEMBL521925	115.00%
CHEMBL522772	20.00%
CHEMBL522936	24.00%
CHEMBL523650	167.00%
CHEMBL524063	4.00%
CHEMBL525772	31.00%
CHEMBL525989	79.00%
CHEMBL526740	146.00%
CHEMBL549746	20.00%
CHEMBL552240	48.00%
CHEMBL556073	30.00%
CHEMBL557580	107.00%
CHEMBL558083	34.00%
CHEMBL559567	20.00%
CHEMBL560160	27.00%
CHEMBL560830	35.00%
CHEMBL562864	59.51%
CHEMBL564036	51.00%

Molecule ChEMBL ID	Relative to Rosiglitazone
CHEMBL564902	24.00%
CHEMBL572565	43.00%
CHEMBL572566	40.00%
CHEMBL572794	42.00%
CHEMBL572795	34.00%
CHEMBL573015	33.00%
CHEMBL573018	50.00%
CHEMBL573252	36.00%
CHEMBL573492	40.00%
CHEMBL574386	39.00%
CHEMBL574606	39.00%
CHEMBL574607	30.00%
CHEMBL574824	26.00%
CHEMBL575687	19.00%
CHEMBL57710	22.15%
CHEMBL578860	50.00%
CHEMBL583300	21.00%
CHEMBL58339	4.48%
CHEMBL583759	27.00%
CHEMBL59132	61.00%
CHEMBL593087	89.13%
CHEMBL593796	95.65%
CHEMBL593801	98.91%
CHEMBL594034	111.96%
CHEMBL595	95.53%
CHEMBL601678	34.00%
CHEMBL602468	31.00%
CHEMBL603	122.51%
CHEMBL603538	60.87%
CHEMBL604491	45.65%
CHEMBL605088	86.96%
CHEMBL60633	31.91%
CHEMBL64972	42.00%
CHEMBL65458	24.00%
CHEMBL66206	55.00%
CHEMBL683	31.00%

Molecule ChEMBL ID	Relative to Rosiglitazone
CHEMBL69018	33.36%
CHEMBL7550	7.61%
CHEMBL86657	91.00%
CHEMBL86658	108.05%
CHEMBL86688	90.00%
CHEMBL86817	161.00%
CHEMBL87307	71.00%
CHEMBL87380	87.00%
CHEMBL87928	112.00%
CHEMBL88917	14.00%
CHEMBL89152	89.00%
CHEMBL89996	85.00%
CHEMBL981	8.67%
